# Unravelling the involvement of cilevirus p32 protein in the viral transport

**DOI:** 10.1038/s41598-021-82453-4

**Published:** 2021-02-03

**Authors:** Mikhail Oliveira Leastro, Juliana Freitas-Astúa, Elliot Watanabe Kitajima, Vicente Pallás, Jesús A. Sánchez-Navarro

**Affiliations:** 1 Unidade Laboratorial de Referência em Biologia Molecular Aplicada, Instituto Biológico, São Paulo, SP Brazil; 2 Embrapa Mandioca e Fruticultura, Cruz das Almas, BA Brazil; 3 Departamento de Fitopatologia e Nematologia, Escola Superior de Agricultura Luiz de Queiroz, Universidade de São Paulo, Piracicaba, SP Brazil; 4 Instituto de Biología Molecular y Celular de Plantas, Universidad Politécnica de Valencia-Consejo Superior de Investigaciones Científicas (CSIC), Valencia, Spain

**Keywords:** Molecular biology, Plant sciences

## Abstract

Citrus leprosis (CL) is a severe disease that affects citrus orchards mainly in Latin America. It is caused by *Brevipalpus*-transmitted viruses from genera *Cilevirus* and *Dichorhavirus*. Currently, no reports have explored the movement machinery for the cilevirus*.* Here, we have performed a detailed functional study of the p32 movement protein (MP) of two cileviruses. Citrus leprosis-associated viruses are not able to move systemically in neither their natural nor experimental host plants. However, here we show that cilevirus MPs are able to allow the cell-to-cell and long-distance transport of movement-defective alfalfa mosaic virus (AMV). Several features related with the viral transport were explored, including: (i) the ability of cilevirus MPs to facilitate virus movement on a nucleocapsid assembly independent-manner; (ii) the generation of tubular structures from transient expression in protoplast; (iii) the capability of the N- and C- terminus of MP to interact with the cognate capsid protein (p29) and; (iv) the role of the C-terminus of p32 in the cell-to-cell and long-distance transport, tubule formation and the MP-plasmodesmata co-localization. The MP was able to direct the p29 to the plasmodesmata, whereby the C-terminus of MP is independently responsible to recruit the p29 to the cell periphery. Furthermore, we report that MP possess the capacity to enter the nucleolus and to bind to a major nucleolar protein, the fibrillarin. Based on our findings, we provide a model for the role of the p32 in the intra- and intercellular viral spread.

## Introduction

Plant virus movement is an active process that may be mediated by one, two or even more encoded movement proteins (MP)^1–4^. MPs facilitate the intracellular viral spread from the viral sites of replication to cell periphery, then to neighboring cells through structural changes in the plant intercellular connections, the plasmodesmata (PD) that correspond to specialized trans-wall channels that allow the transport of small molecules between the cytoplasm of neighboring cells. Once the MP reaches the PD, this protein may assist the virus transport to neighboring cells as a viral ribonucleoprotein (vRNP) complex, viral replication complex (VRC)^5–7^ or as an entire virus particle through the formation of tubular structures by MP polymerization. An intermediated category is suggested, where tubular structures promote the movement of RNPs rather than entire particles^4,8–10^. Viral intercellular transport in plants encompasses two main mechanisms well described in the literature^11–13^. The tubule-guided mechanism results in the formation of tubular structures that drastically modify the PD and facilitate the virus passage in the form of virions^10^. On the other hand, the non-tubule-guided mechanism encompasses MPs that are capable to interact with the viral RNA and facilitate the transport of a vRNPs through the PD without causing any visual changes^12,14,15^. Other viruses require the CP for virus transport but as part of the vRNP, where the formation of virus particles is not a prerequisite for virus translocation^8,16^. For viral systemic movement, plant viruses are passively transported to distant regions of the plant through the vascular tissue (phloem) in different biological forms: virions with their genome protected by capsid protein assembled or as RNP complexes containing the viral genome associated with viral and/or host proteins^17,18^. Given the complexity of the molecular mechanisms governing the virus long-distance spread, very little has been explored about systemic mechanism of viral movement so far, which represents a challenge for virologists.

Citrus leprosis complex is considered a major viral disease affecting citrus orchards distributed from South to North America. This re-emergent disease is caused by *Brevipalpus*-transmitted viruses (BTV) that exhibit significant differences in their genomic organization and in the cellular infection process. The BTVs belonging to the genus *Cilevirus,* family *Kitaviridae* have a positive single stranded RNA (_(+)_ssRNA) and replicate in the cell cytoplasm; in contrast, the species belonging to the genus *Dichorhavirus,* family *Rhabdoviridae* with a negative single stranded RNA (_(−)_ssRNA) genome, replicate into the cell nuclei^19,20^. Currently, citrus leprosis virus C (CiLV-C), the type member of the genus *Cilevirus,* is considered the most devastating virus infecting citrus in South American countries, especially in Brazil^21^. However, this situation has been changing, in Colombia, where CiLV-C, prevalent ten years ago, is now rarely found, with the increased incidence of citrus leprosis virus C2 (CiLV-C2)^19,22–24^. The natural infection by BTV results only in localized lesions; for a yet unknown process, BTVs seem to be unable to reach phloem tissues, thus apparently unable to become systemic in their hosts.

Previous prediction studies have revealed that the p32 ORF, located in the RNA2 of CiLV-C, shares conserved motifs with the 30K superfamily of MPs^25,26^, including the seven predicted beta-strands connected by putative loops with different patterns of sequence conservation and the aspartic acid residue or “D motif” at the end of the strand 3^27^. In a recent study, we described the capacity of p32 protein of CiLV-C to traffic along the endoplasmic reticulum (ER) system and its co-localization with the plasmodesmata (PD), suggesting its putative role in cell-to-cell movement^28^. Although putative MPs have been indicated for cileviruses, a biological study about the protein performance on aspects involved with viral spread is lacking. In this context, we have analyzed the MP functions of two species associated with the citrus leprosis disease: the cileviruses CiLV-C strain CRD and CiLV-C2, using a heterologous alfalfa mosaic virus (AMV) model system, which allows the functional exchange of MPs assigned to the “30K superfamily”^9,29–31^.

Our results provide robust evidence that p32 represents the movement protein of cileviruses. We studied the function of this MP in various aspects related to cell-to-cell and systemic movement. In addition, we demonstrated the capacity of MP to redirect the viral capsid protein from their site of accumulation in the cytoplasm to cell periphery, suggesting the formation of a MP-CP cis-interaction complex. We have further demonstrated the nucleolar localization of MP and its interaction with the fibrillarin. Based on all our findings, we propose that encoded p32 is the viral movement protein, and more importantly, we present a model for the role of the cileviruses MP in viral spread.

## Results

### The cileviruses MP is able to complement the cell-to-cell and systemic AMV transport and is not dependent of the CP interaction for these processes

**Figure 1:**
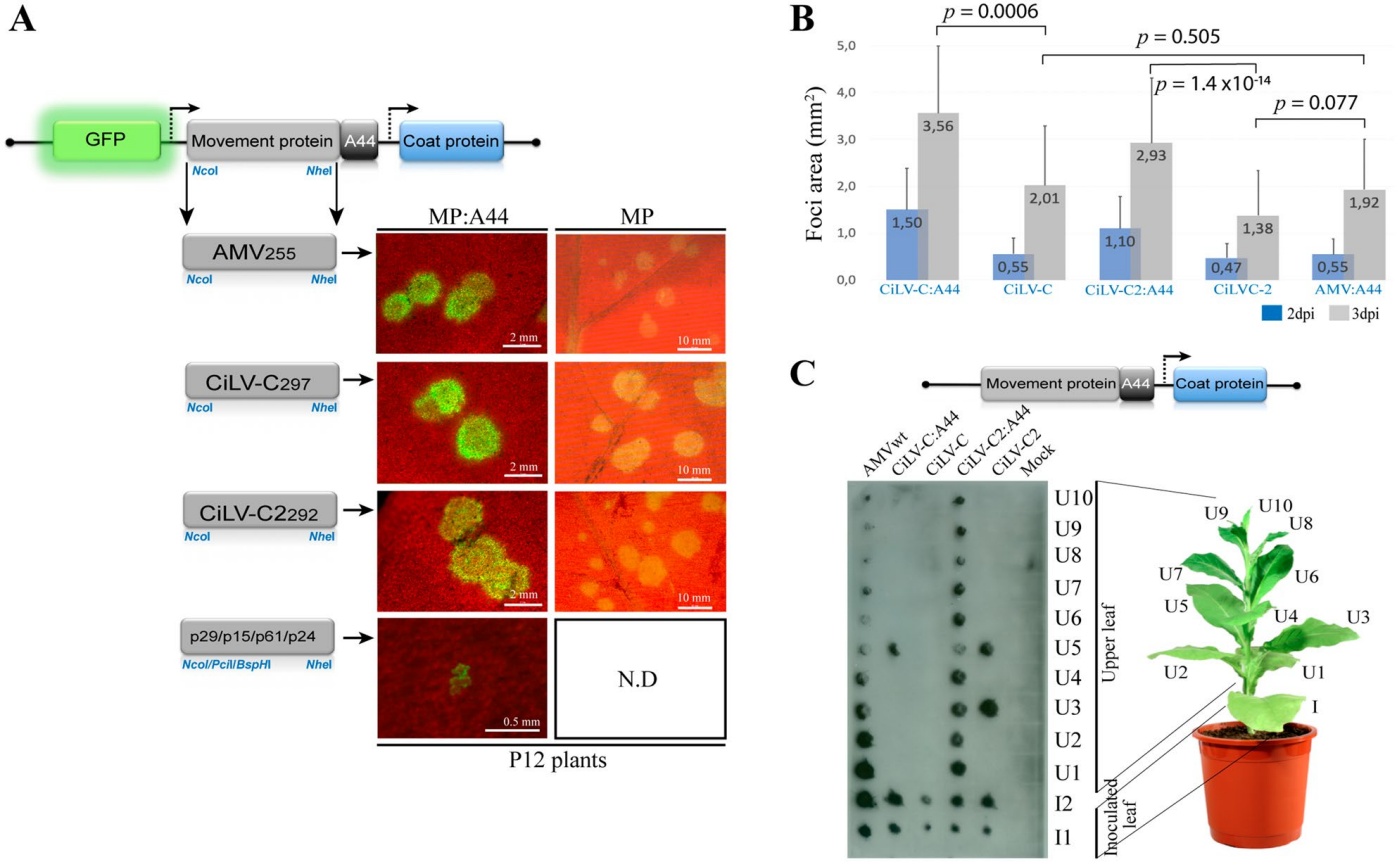
The cileviruses MPs complement the cell-to-cell and systemic movement of the AMV infectious clone. Analysis of the cell-to-cell and systemic transport of the hybrid AMV RNA 3 in which its MP gene was exchanged with the corresponding genes (MPs) of CiLV-C and CiLV-C2 and the genes p29, p15, p61 and p24 of CiLV-C. (**A**) Infection foci observed in P12 plants inoculated with RNA 3 transcripts from pGFP/A255/CP derivatives carrying the AMV MP lacking the C-terminal 44 residues (A44) (MP), the AMV MP wild type (MP:A44), and the aforementioned heterologous MPs alone (right panels; MP) or fused to A44 (left panels; MP:A44). The schematic representation shows the GFP/A255/CP AMV RNA 3^29^, in which the open reading frames, represented by large boxes, correspond to the green fluorescent protein (GFP), the movement protein (MP) and the coat protein (CP). Short box corresponds the C-terminal 44 amino acids of the AMV MP, meanwhile arrows represent subgenomic promoters. The numbers after the viral acronym represent the total amino acids residues of the corresponding MP. The *Nco*I and *Nhe*I restriction sites used for insertions of the MPs are indicated, as well as the restriction sites *BspH*I, *Pci*I and *Nhe*I for insertions of the other CiLV-C genes. White bars represent to 500 μm, 2 mm and 10 mm. *N.D* non-determined. (**B**) Histograms represent the average of the area in mm^2^ of 80 independent infection foci at 2 and 3 days post-inoculation (dpi). Error bars indicate the standard deviation. Student’s *t*-test and significance was set at *p* < 0.05. The *p*-values obtained from comparison between pairs of groups are presented. (**C**) Tissue-printing analysis of P12 plants inoculated with the AMV RNA 3 derivatives showed in A but lacking the 5′ proximal GFP gene. Plants were analyzed at 14 dpi by printing the transversal section of the corresponding petiole from inoculated (I) and upper (U) leaves. Mock corresponds to non-inoculated plant.

In a previous study, we found the p32 protein co-localized with plasmodesma structures at cell periphery^28^, suggesting that this protein could play a role in viral movement. Here, we evaluate if cileviruses MPs are able to complement the cell-to-cell transport of AMV. The MPs of CiLV-C and CiLV-C2 were inserted into the AMV RNA3 infectious construct that expresses the GFP (pGFP/A255/CP)^29^ by exchanging the AMV MP. Two variants of this AMV construct were tested, which differed in the presence or absence at the C-terminus of the heterologous MP, of the C-terminal 44 amino acids (A44) of the AMV MP, a region required for a compatible interaction with AMV coat protein (CP)^9^. In vitro transcripts from these heterologous constructs were mechanically inoculated in leaves of *Nicotiana tabacum* plants that constitutively express the P1 and P2 subunits of the AMV replicase (P12 plants), to evaluate the cell-to-cell movement and compared to the AMV wild type (wt). The P12 plants permit to work only with the AMV RNA 3 or its derivatives, simplifying the analysis. All constructs harboring the A44 residues, resulted in clear fluorescence infection foci at 2 dpi (Fig. 1A, left panels), indicating that the cileviruses MPs are able to complement the cell-to-cell movement of the chimeric construct. The analysis of the area of 80 independent infection foci at 3 dpi revealed bigger infection foci derived from the construct carrying the CiLV-C:A44 MP (average of 3.56 mm^2^) followed with the CiLV-C2:A44 derivative (2.93 mm^2^) and the AMV wt (1.92 mm^2^) (Fig. 1B). The constructs lacking the A44 residues were also competent for the cell-to-cell transport (Fig. 1A, right panels), but significantly less efficient than those harboring the A44 (*p-*value < 0.05). Thus, we observed a decrease of 1.55 mm^2^ in the foci area comparing the CiLV-C:A44 and CiLV-C constructs (3.56 mm^2^ versus 2.01 mm^2^) and 1.55 mm^2^ between the CiLV-C2:A44 and CiLV-C2 derivatives (2.93 mm^2^ versus 1.38 mm^2^), at 3 dpi (Fig. 1B). However, both constructs carrying the heterologous MPs non-fused to A44 (CiLV-C or CiLV-C2) generated infection foci similar to those caused by the AMV wt (Fig. 1B), and the *t*-test did not indicate significant differences at 3 dpi (*p-*value > 0.05). The observation that both cilevirus MPs were competent for the cell-to-cell transport of AMV, regardless of the fusion of the A44 region, suggests that both proteins could mediate the AMV transport independently of the AMV CP interaction. To discard a putative interaction between cilevirus MPs and the AMV CP, we performed an in vivo protein interaction BiFC assay. Reconstitution of the YFP fluorescence was not detected in all protein pair combinations performed (Fig. S1), suggesting non-heterologous interactions.

Further analyses were addressed to evaluate the capability of the cileviruses MP to support the systemic movement of AMV RNA 3. To do this, we used an AMV RNA3 wild-type clone that does not express GFP (pAL3NcoP3), since the RNA 3 derivatives harboring the GFP reporter gene do not support systemic movement in P12 plants^29^. The distribution of the RNA 3 expressing the heterologous MPs in inoculated and systemic P12 leaves was analyzed by tissue-printing of petiole cross sections, where positive hybridization signal correlated with the presence of the virus in the corresponding leaf, as previously described^30–32^. We observed the presence of the viral RNA in inoculated leaves (I) and upper leaves (U) for all heterologous construction expressing the A44 fragment and AMV control (Fig. 1C; lines 1, 2 and 4), indicating that the cilevirus p32 is a movement protein also able to complement the systemic movement of the AMV RNA 3 derivative. The chimeric constructs expressing the MP of CiLV-C2:A44 (Fig. 1C, line 4) showed the same dispersion pattern of the AMV control (Fig. 1C, line 1) with the presence of viral RNA in all upper leaves (U1 to U10 in Fig. 1C); on the other hand, the CiLV-C:A44 MP was less efficient to support the systemic spread, being able to infect just the inoculated and the upper leaf 5 (U5) or 2 (U2) (Fig. 1C, line 2; Fig. 3D, line 1) or the first 5 upper leaves (data not shown) but never all upper leaves, as observed with the AMV and CiLV-C2 MPs, indicating a limitation of systemic spread.

When the same experiment was performed with the AMV RNA 3 constructs expressing the heterologous MPs non-fused to the A44, CiLV-C MP was not able to support the systemic transport of the hybrid AMV virus, resulting in virus detection only on inoculated leaves (Fig. 1C, line 3). Unlike CiLV-C MP, the construct expressing the CiLV-C2 MP was able to support the systemic transport regardless its fusion with the A44 residues, since the virus was detected in the inoculated and in the U3 and U5 upper leaves (Fig. 1C, line 5 and consistent in all replicates), indicating that this MP allows the systemic AMV transport independently of its AMV CP interaction.

In order to evaluate if other proteins encoded by cileviruses have the ability to complement the AMV viral movement, we inserted the p29, p15, p61 and p24 CiLV-C genes into the AMV RNA 3 constructs harboring the GFP reporter gene. In all cases, only single fluorescent cells were observed on P12 leaves (Fig. 1A), indicating the inability of these proteins to complement the cell-to-cell transport of the AMV RNA 3 derivatives.

### The intercellular spread of cileviruses is independent on nucleocapsid assembly

**Figure 2:**
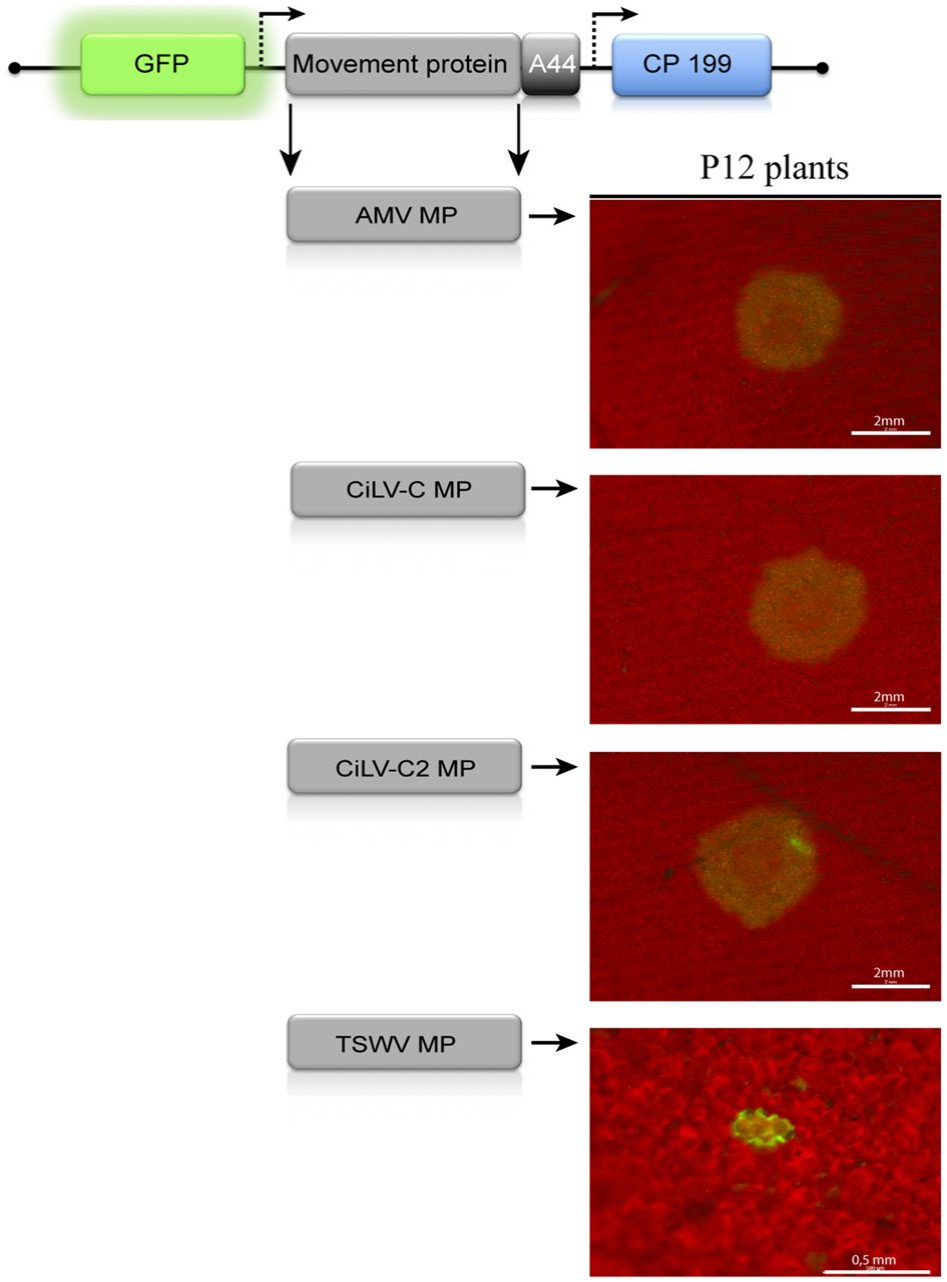
The cilevirus MP is able to transport infectious viral complexes. Analysis of cell-to-cell transport of the hybrid AMV RNA3, carrying different MP genes and the mutated coat protein gene (CP 199) defective in virus particles formation. The schematic representation corresponds to the same constructs indicated in Fig. 1A, in which the CP gene was replaced by the CP 199 gene from the construct pGFP/BMV:A44/CP199^9^ by exchanging the *Nhe*I-*Pst*I fragment. P12 plants were inoculated with transcripts derived from the AMV RNA 3 variants expressing AMV wt (positive control), CiLV-C, CiLV-C2 and TSWV (negative control) MPs. The images of foci formation or single-cell GFP foci were observed at 2 days post-inoculation (dpi) using a Leica Stereoscopic Microscope. Each infection foci image is representative of the inoculation of three leaves per plant and three plants inoculated for each chimeric AMV construct. White bars represent 0.5 mm to 2 mm.

To obtain additional insights about the performance of the cileviruses MP in viral transport, we examined whether the viral complex transported intercellularly by these MPs is dependent on the virus particle formation. For this purpose, we performed a deletion of C-terminal 21 amino acids of the coat protein (CPN199) on the pGFP/MP:A44/CP chimeric construct. It has been previously demonstrated that an infectious clone expressing the CPN199 is competent for cell-to-cell spread and RNA accumulation, but not for virion encapsidation^33^. Clear infection foci were visualized with the two AMV constructs carrying the cilevirus MPs and the CPN199 (Fig. 2), similar to what is observed for the AMV wt (positive control). The AMV construct carrying the NSm movement protein of tomato spotted wilt orthotospovirus (TSWV; negative control), that was able to transport only encapsidated virions^30^, generated single fluorescent cells (Fig. 2). These results demonstrate that the virus particles are not required for the cell-to-cell transport mediated by cileviruses MPs.

### The C-terminal region of cileviruses MP is dispensable for cell-to-cell movement, but its expression is essential for an efficient transport

**Figure 3:**
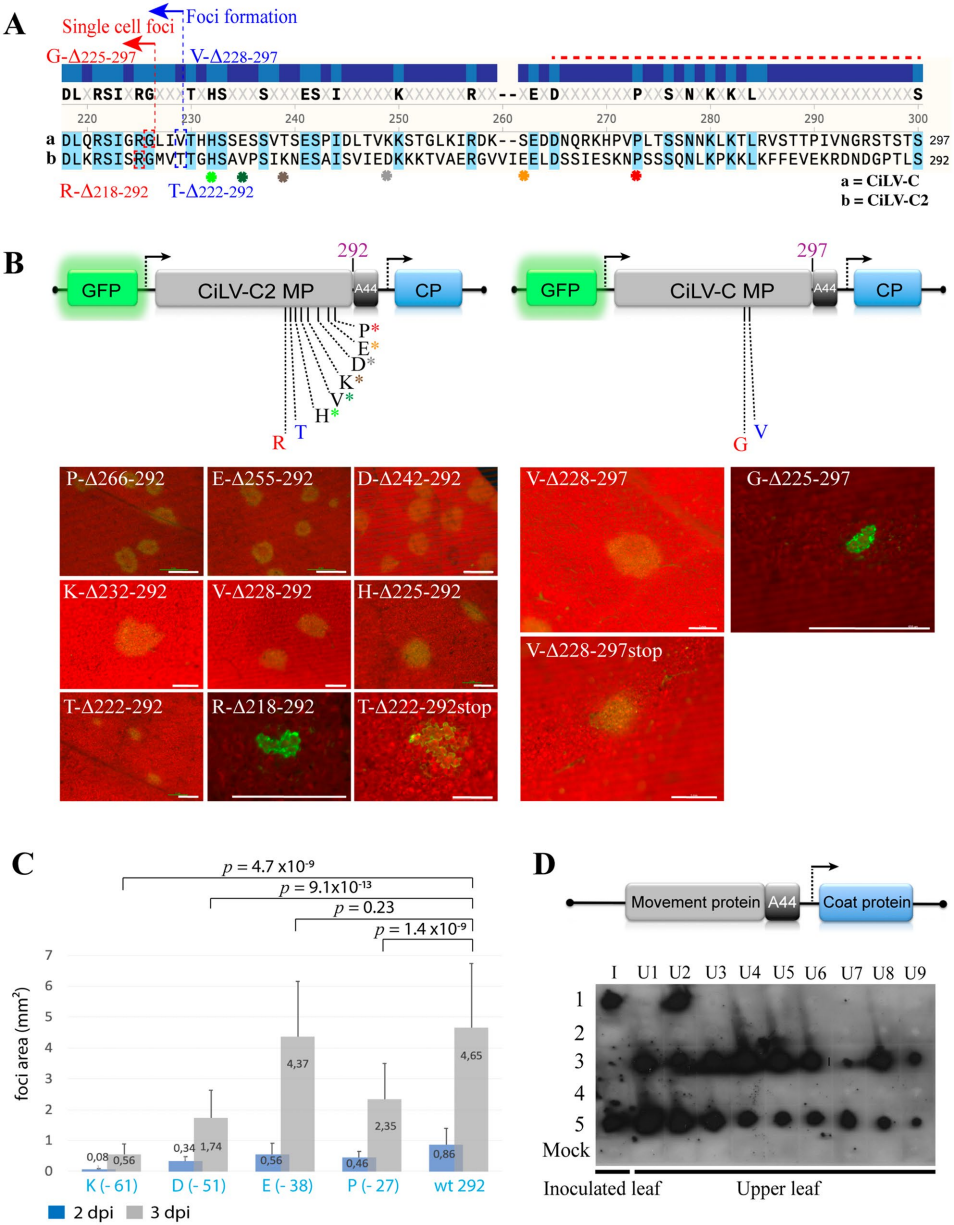
The C-terminus of cileviruses MP is dispensable for cell-to-cell movement. (**A**) Alignment of amino acids sequence of the C-terminal region of CiLV-C and CiLV-C2 MPs. Residues common to the two sequences are shown in blue. Red dotted line indicates the most variable region at the C-termini, corresponding the last 36 residues of the MP. The alignment was performed using the software SnapGene 4.3.10. (**B**) Infection foci observed in P12 plants inoculated with RNA 3 transcripts carrying the C-terminal truncated MP genes of cileviruses. The schematic representation shows the chimeric GFP/A255/CP AMV RNA 3 represented in Fig. 1A and the C-terminal deletions of the MP proteins analyzed. Amino acid numbers 292 and 297 correspond to wild-type (wt) size of the corresponding MP proteins, meanwhile residues P, E, D, K, V, H, T and R for CiLV-C2 and V, G for CiLV-C correspond to the last amino acid included in the corresponding truncation. Superscript asterisks refer to the residues shown in alignment (**A**) correlated by their respective colors. Each infection foci image is representative of the inoculation of three leaves per plant and three plants inoculated for each chimeric AMV construct at 2 dpi. White bars correspond to 200 μm. (**C**) Histograms represent the average of the area in mm^2^ of 80 independent infection foci at 2 and 3 dpi of the P12 plants infected with hybrids AMV RNA 3 containing the wild type and truncated CiLV-C2 MPs P-Δ_266–292_, E-Δ_225–292_, D-Δ_242–292_ and K-Δ_232–292_ lacking -27, -38, -51 and -61 residues, respectively. Error bars indicate the standard deviation. Student’s *t*-test and significance was set at *p* < 0.05. The *p*-values obtained from comparison between pairs of groups are presented. (**D**) Tissue-printing analysis of P12 plants inoculated with a variant of AMV RNA 3 expressing the CiLV-C wt (297)(1), CiLV-C Δ_228–297_ (V)(2), CiLV-C2 wt (292)(3), CiLV-C2 Δ_222–292_ (T)(4) and AMV MP (AMV)(5). Plants were analyzed at 14 dpi by printing the transversal section of the corresponding petiole from inoculated (I) and upper (U) leaves.

A common property observed for C-terminal region of the MPs assigned to the 30K superfamily was its dispensability for the cell-to-cell spread^8,9,30,34–36^. Given this context, we analyzed if such property could also be applied to cilevirus MPs. Firstly, we aligned the MPs of CiLV-C and CiLV-C2, observing a high variability at the C-terminal region (between amino acids 160 and 297) (see complete alignment in Fig. S3), with most divergence located in the last C-terminal 36 residues (Fig. 3A, red dotted line). From this analysis, sequential C-terminal deletions of the CiLV-C2 MP were performed until the protein was not competent to support the virus movement. For this purpose, a chimeric RNA 3 AMV harboring GFP reporter gene, was used to evaluate the functionality of the truncated cileviruses MP. C-terminal mutants of the CiLV-C2 MP revealed that deletions up to 70 residues generated MPs competent to support the cell-to-cell movement of the chimeric AMV RNA3 in P12 plants, showing clear fluorescent infection foci (Fig. 3B, T-Δ_222–292_). However, when the C-terminal 74 amino acids (aa) were deleted (construct R-Δ_218–292_), only single fluorescent cells were visualized (Fig. 3B), indicating that residues located between R-T (“GMV”, see Fig. 3A) seem to be essential for cell-to-cell spread. The same pattern was observed using the CiLV-C MP (Fig. 3B), in which deletion of the C-terminal 69 aa (construct V-Δ_228–297_) generated a MP competent for the cell-to-cell transport meanwhile deletion of three additional residues (construct G-Δ_225–297_) rendered a nonfunctional MP. In the next step, we evaluated the efficiency of the functional cilevirus MPs lacking different portions of the C-terminus. To do that, we measured the infection foci area at 2 and 3 dpi of the AMV RNA3 chimeric construct carrying the CiLV-C2 MP wt or its derivatives lacking the C-terminal 27 (P-Δ_266–292_), 38 (E-Δ_255–292_), 51 (D-Δ_242–292_) and 61 (K-Δ_232–292_) residues, respectively. The analysis of the area of 80 independent infection foci revealed a significant decrease (*p-*value < 0.05, except for mutant E-Δ_255–292_), mostly evident in a comparison between the MP wt with the construct lacking 61 residues (K-Δ_232–292_), rendering infectious foci with an area representing 12% of MP wt (average of 0.56 mm^2^ versus 4.65 mm^2^ at 3 dpi) (Fig. 3C). This finding reveals that although the movement capacity of MP mutants lacking different C-terminal portions is maintained, the removal of this region affects considerably the efficiency of the cell-to-cell transport.

Next, we evaluated whether the large cilevirus MPs C-terminal mutants functional for the cell-to-cell transport were still able to support the systemic movement. To do this, the CiLV-CΔ_228–297_ and CiLV-C2Δ_222–292_ MP mutants lacking the C-terminal 69 and 70 residues, respectively were assayed in the AMV RNA3 wt construct. Tissue-printing analysis reveals no hybridization signal in the petiole of inoculated and all upper P12 leaves, meanwhile the control AMV and the cilevirus MPs wt showed the viral presence in inoculated and upper leaves, as expected (Fig. 3D).

### The C-terminal region of MP is necessary for tubule formation and correct association with the plasmodesma

To further characterize the cilevirus MPs, we investigated first the polymerization of tubule structures on the surface of the *Nicotiana benthamiana* protoplasts. To do this, the MPs carrying a C-terminal eGFP fusion, were transiently expressed in *N. benthamiana* leaves by agroinfiltration. The protoplasts were purified 24 h post-inoculation (hpi) and the eGFP signal was visualized 16 h after purification. All cilevirus MPs induced the formation of tubular structures on the protoplast periphery (Fig S2a,b), regardless of viral infection. This indicates tubule-forming property for cilevirus MPs.

**Figure 4:**
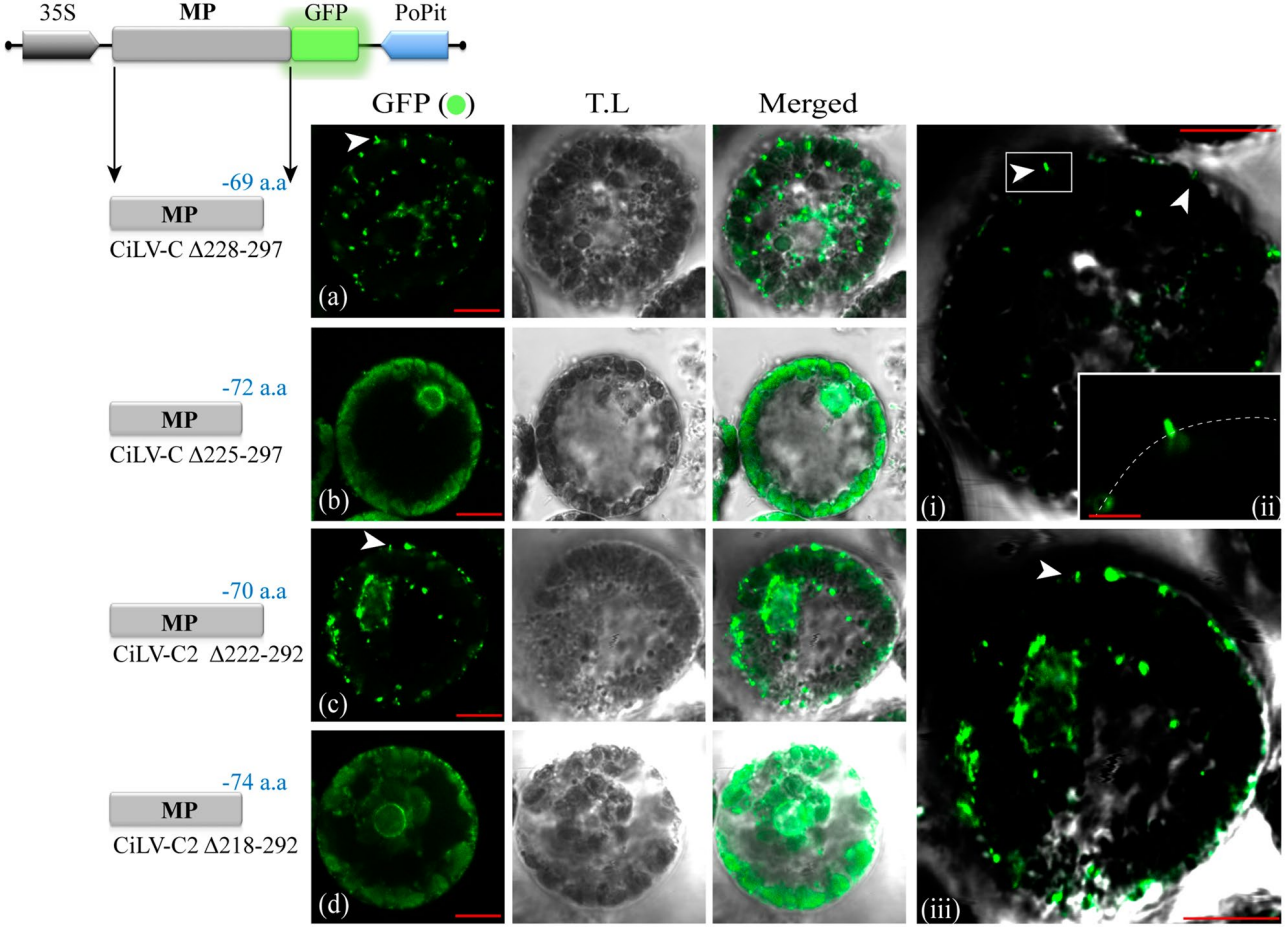
The C-terminal of the cileviruses MP is intrinsically related to the tubule formation. Analysis of tubule formation from transient expression of the truncated MP proteins of the CiLV-C and CiLV-C2 fused at their C-termini with eGFP (green filled circle) on the surface of *N. benthamiana* protoplasts. Three infiltrated leaves per construct were used for protoplasts isolation. Protoplasts were purified after 1 day post-infiltration and the fluorescence GFP signal was captured 16 h post-protoplasts purification with a Zeiss LSM 780 confocal laser-scanning microscope. Each image-frame expressing GFP represents the visualization of several protoplasts (about 15 to 20) per assay for each MP construct analyzed. The green (GFP), transmitted light (TL) channels and merged images are shown. The scheme indicates the expression cassette introduced into the binary vector pMOG_800_ with the 35S cauliflower mosaic virus promotor region (black arrow box), the MP gene (gray box) carrying the eGFP report gene (green box) fused at their C-termini and the potato proteinase inhibitor II terminator region (PoPit) (blue arrow box). The number of deleted residues for each truncated MP is shown in blue. Short tubules are visualized for the MP truncated versions with maximum limit of deletions in movement functions still able to generate cell-to-cell spread (CiLV-C Δ_228–297_ and CiLV-C2 Δ_222–292_), while no tubules were observed for the constructions unable to generate movement (CiLV-C Δ_225–297_ and CiLV-C2 Δ_218–292_). The panels i–iii shown merged images of protoplasts highlighting the presence of short tubules (white arrows). T.L brightness was lowered to facilitate the visualization of short tubules. In panel ii, the dotted line simulates the cell periphery. Red bars correspond to 5–10 μm.

In the next step, we analyzed if the negative effects in virus transport observed with the MPs lacking the C-terminal could be related to the capacity to generate tubular structures on protoplasts. To this end, the truncated versions of MPs: CiLV-CΔ_228–297_, CiLV-CΔ_225–297_, CiLV-C2Δ_222–292_ and CiLV-C2Δ_218–292_, carrying a C-terminal eGFP were assayed in protoplasts as aforementioned. The constructs that generated infectious foci with low movement efficiency (CiLV-CΔ_228–297_ and CiLV-C2Δ_222–292_) showed the GFP signal accumulation in punctuated structures dispersed along the protoplast periphery with the formation of short tubules (see white arrows in Fig. 4a,c and panels i–iii) when compared with wt MPs constructs (Fig. S2); on the other hand, the non-functional constructs in viral transport (CiLV-CΔ_225–297_ and CiLV-C2Δ_218–292_) rendered fluorescent dots and diffuse signal at the protoplast surface but not tubular structures (Fig. 4b,d). These results clearly indicate that the C-terminal region of both MPs influence the tubule formation.

**Figure 5:**
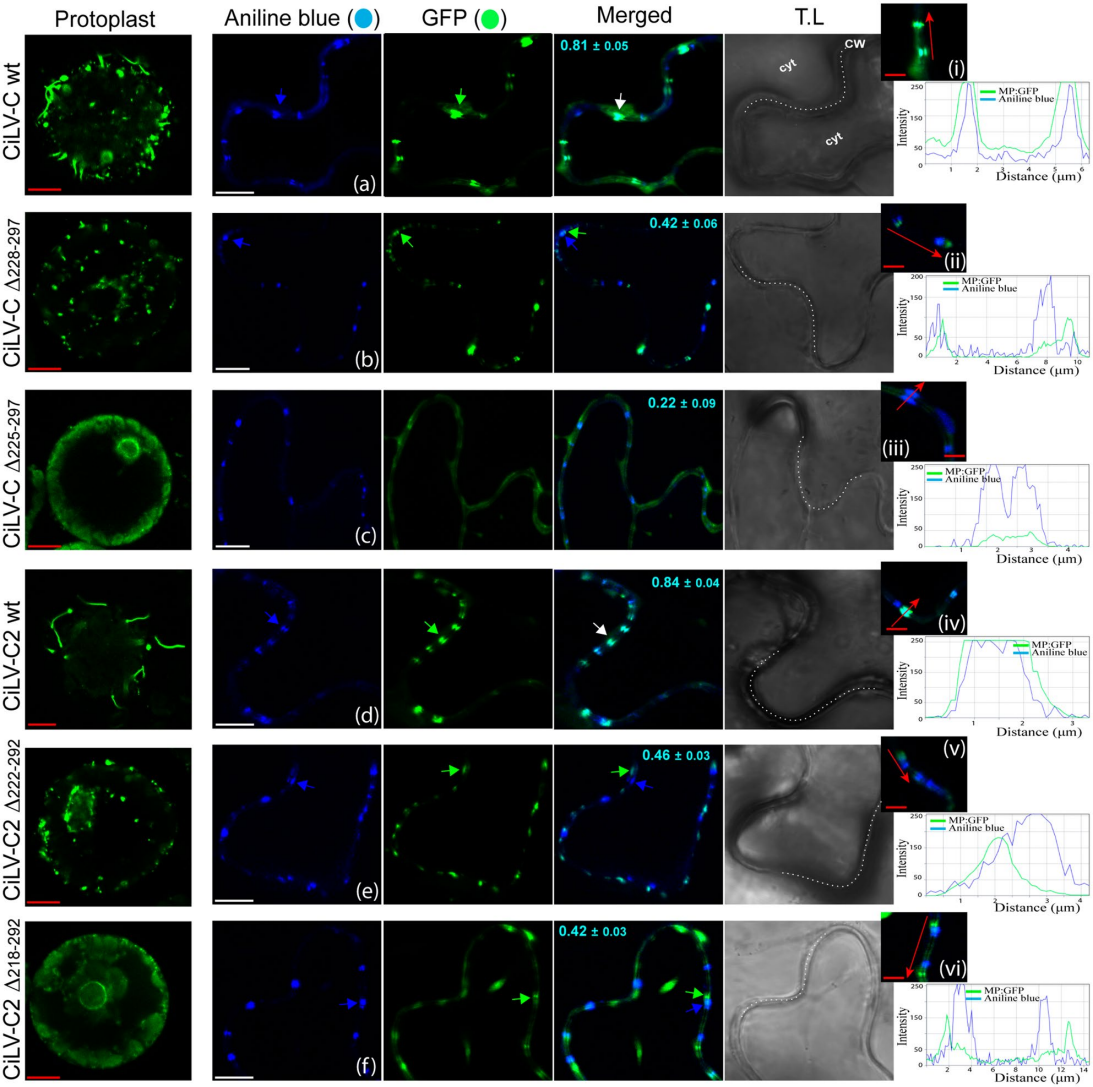
The C-terminal region of cileviruses MP is necessary for correct co-localization with plasmodesmata. Transient expression of CiLV-C wild type (**a**), CiLV-CΔ_228–297_ (**b**), CiLV-CΔ_225–297_ (**c**), CiLV-C2 wild type (**d**), CiLV-C2Δ_222–292_ (**e**) and CiLV-C2Δ_218–292_ (**f**) MPs, carrying the GFP (green filled circle) in *N. benthamiana* leaves. Fluorescence signal was captured at 72 h post-infiltration. The callose deposits were stained using aniline blue (blue filled circle). The blue and green arrows indicate the callose deposits in plasmodesma and MP, respectively; while the white arrows indicate co-localization between them. The blue (aniline blue), the green (GFP), transmitted light (TL) channels and merged images are shown in the figure. Images on the left show the tubule formation of *N. benthamiana* protoplast transiently expressing the correspondent MP construct. The MP punctate structures co-localize at the cell periphery with the fluorochrome for the MP wt constructs (**a,d**), while the truncated MP constructs show no co-localization or partial co-localization (**b,c,e,f**). In a higher magnification image, it is shown the complete (i,iv) or partial (ii,iii,v,vi) co-localization of the MP with callose deposits in the plasmodesmata, and the chart of fluorescence intensities further confirms the co-localization. Plot shows green and blue fluorescence intensities, indicated by MP:GFP and aniline blue, in the selected region of interest (red arrows). Distance measurement starts from the base to the tip of the arrows (x axis). The mean SD of Person Correlation Coefficient (PCC) is given in the merged image. PCC was measured using the Fiji co-localization plugin for three independent images from approximately 100 individual plasmodesmata. The dotted line in the transmitted light image indicates the cell wall (CW). *Cyt* cytoplasm. Red and white bars correspond to 5–10 and 20 μm, respectively.

In the next step, we evaluated the implication of the C-terminal region in the MP association with plasmodesmata. To do that, C-terminal MP deletion constructs carrying the GFP fused at its C-terminus were transiently expressed in *N. benthamiana* leaves. After staining the plasmodesmata (PD) structures with a callose marker (aniline blue), we observed that deletion of 69 residues of the CiLV-C MP (CiLV-CΔ_228–297_) impairs the correct co-localization (Person Correlation Coefficient [PCC] = 0.42) of the MP with PD (see arrows, Fig. 5b) when compared with MP wt construct (Fig. 5a, PCC = 0.81). Deletions of 72 residues of the CiLV-C MP (CiLV-CΔ_225–297_) completely impaired the interaction with PD (PCC = 0.22), resulting in a GFP diffuse signal along the plasma membrane without the formation of punctate structures at the cell periphery (Fig. 5c). The same disturbance was observed using the CiLV-C2 MP, where the C-terminal mutant lacking 70 residues (CiLV-C2Δ_222–292_) rendered a PCC co-localization value of 0.46, less than that observed with the MP wt (PCC = 0.84) (Fig. 5e,d, respectively). However, and unlike the CiLV-C MP, the GFP signal derived from the construct CiLV-C2Δ_218–292,_ rendered a fluorescent punctate signal along the cell periphery (Fig. 5f) similar to that observed for the CiLV-C2Δ_222–292_ MP mutant (Fig. 5e), but with higher disturbance on the interaction with PD (PCC = 0.42), compared with the CiLV-C2Δ_222–292_ construct. The co-localization of CiLV-C and CiLV-C2 wt MPs and the corresponding truncated versions with plasmodesmata was further analyzed by measurements of fluorescence intensity across the plasma membrane. Fluorescent GFP signal of CiLV-C and CiLV-C2 wt MPs coincide with all callose marker signal (Fig. 5ai,div); on the other hand, GFP signal derived from the CiLV-CΔ_228–297_, CiLV-CΔ_225–297_, CiLV-C2Δ_222–292_ and CiLV-C2Δ_218–292_ constructs did not match completely with plasmodesma signal along the plasma membrane (Fig. 5b ii,ciii,ev,fvi), further suggesting that these deletions alter the correct localization of the MP to the plasmodesma structures. To see this effect clearly, we decided to determine the percentage of callose-stained PD which co-localize to the MP-GFP fusion proteins (Table 1). Thus, the CiLV-C and CiLV-C2 MPs wt were detected in the 92.3% and 93.7% of stained PD, respectively, meanwhile C-terminal MP mutants lacking the C-terminal 69 (CiLV-CΔ_228–297_), 72 (CiLV-CΔ_225–297_), or 70 (CiLV-C2Δ_222–292_), 74 (CiLV-C2Δ_218–292_) residues were detected in 45.3%, 9.7% or 48.2%, 34.7% of PD, respectively, indicating that the C-terminal region of cilevirus MPs has a positive role in the MP-PD co-localization.

**Table 1:** Percentage of plasmodesmata which colocalize with cilevirus MPs, p29 and MP-p29 complexes.

	Colocalized with PD	Total PD	% value
CiLV-C MP_wt_:GFP + Ab	97	105	92.38
CiLV-C MPΔ_228–297_:GFP + Ab	52	115	45.22
CiLV-C MPΔ_225–297_:GFP + Ab	11	113	9.73
CiLV-C MPΔ_1–227_:GFP + Ab	0	108	0.00
CiLV-C2 MP_wt_:GFP + Ab	119	127	93.70
CiLV-C2 MPΔ_222–292_:GFP + Ab	55	114	48.25
CiLV-C2 MPΔ_218–292_:GFP + Ab	40	115	34.78
p29_wt_:GFP + Ab	8	96	8.33
p29_wt_:GFP + MP_wt_:HA + Ab	55	94	58.51
p29_wt_:Nt + MPΔ_1–227_:Ct + Ab	31	108	28.70
p29_wt_:Nt + MPΔ_228–297_:Ct + Ab	0	100	0.00

*Ab* aniline blue (callose staining), *PD* Plasmodesma, *wt* wild type, *Nt* N-part of the enhanced yellow fluorescent protein, *Ct* C-part of the enhanced yellow fluorescent protein.

### Cileviruses MP interact with the cognate capsid protein (p29) through both N- and C-terminal regions

Previous results showed that the MP of CiLV-C is able to interact in vivo with the cognate capsid protein^28^. In the next step, we decided to determine by BiFC analysis whether or not the characterized C-terminal region of the cilevirus MPs, dispensable for viral transport, is required to interact with the cognate cilevirus CPs. To do that, wild-type (CiLV-C MP wt and CiLV-C2 MP wt) and mutated MPs lacking the C-terminus (CiLV-C MPΔ_228–297_ and CiLV-C2 MPΔ_222–292_) or the remaining N-terminus (CiLV-C MPΔ_1–227_ and CiLV-C2 MPΔ_1–221_) were co-expressed with their cognate p29 proteins. Reconstitution of the YFP fluorescence was detected in all MPs versions co-expressed with the p29 proteins (Fig S4Aa–f) suggesting positive interactions, whereas no fluorescence was observed when the viral MPs were co-expressed with the unfused CYFP (CYFPcyt) or with the counterpart (C-YFP) targeted to the ER (CYFPer), (Fig. S4Ag–i). We also used the nucleocapsid (N) of TSWV, a virus evolutionarily distinct from cileviruses, which was expressed in combination with the cilevirus MPs. In all cases we did not observed reconstitution of the YFP fluorescence (Fig. S4Ag–i). Co-immunoprecipitation (Co-IP) experiments between CiLV-C and CiLV-C2 MPs and the respective cognate p29 proteins confirmed the MP-p29 interactions (Fig. S4B). Taken together, these results characterize the interaction of MP and p29 and suggest the possible presence of two or more regions of the cilevirus MPs with capacity to interact with the p29 protein.

### The MP co-expressed with p29 redirects the p29 from the cytoplasm to cell periphery associating with the plasmodesmata

**Figure 6:**
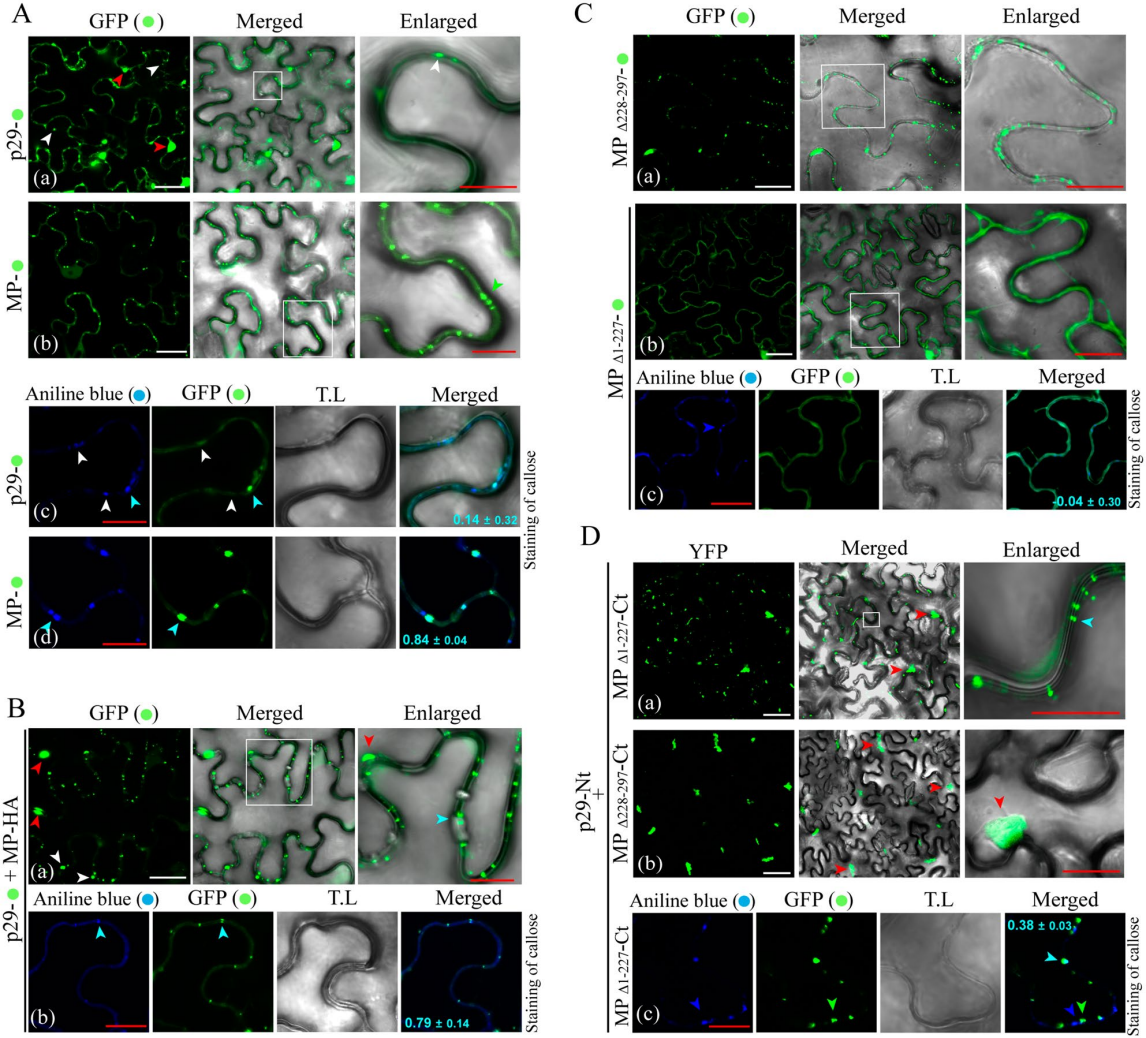
Redistribution of the coat protein (p29) from the cytoplasm to callose deposits in plasmodesma by interaction with the MP of CiLV-C. (**A**) Subcellular localization of the CiLV-C p29 and MP fused with eGFP (green filled circle) transiently expressed in *N. benthamiana* leaves visualized at 72 h post-infiltration. White boxes correspond to high magnification to highlight callose deposits. White and red arrows indicate small punctate structures and large agglomerates of p29 (a). The green arrow in enlarged image in (b) indicates MP punctate structures along the cell membrane. (c,d) show the callose deposits stained with aniline blue (blue filled circle). White and blue arrows indicate regions of callose deposition at the cell membrane and the co-localization among the callose deposits in plasmodesma with the p29 or MP proteins, respectively. (**B**) Expression assay to determine the re-co-localization of the p29 when co-expressed with the MP. The p29-eGFP was co-expressed with the MP-HA (a). Red and white arrows indicate p29 large and small structure, respectively. The callose staining (b) shows the co-localization between the p29 with callose deposits in plasmodesmata (blue arrow). (**C**) Sub-cellular localization of the mutated MP CiLV-C MPΔ_228–297_ (a) lacking the C-terminus, and the remaining N-terminal region (CiLV-C MPΔ_1–227_) (b), also stained with aniline blue fluorochrome (c). (**D**) BiFC analysis to determine the MP region responsible for redirecting the coat protein. The C- and N-fragment of MP (a/c,b, respectively) fused at their C-terminus with CYFP were co-expressed with the cognate p29 protein fused at its C-terminus with the NYFP counterpart. Red and blue arrows indicate agglomerates dispersed throughout the cytoplasm and punctate structures along cell periphery, respectively. In panel c, the dark blue and green arrows indicate the callose deposits and MP, respectively; while the light blue arrow indicates co-localization between them. The mean SD of PCC is given in the merged image that shows callose staining. The BiFC fluorescence was visualized at four days pot-infiltration. The blue (aniline blue), the green (GFP), transmitted light (T.L) channels and merge images are shown in the figure. White and red bars correspond to 50 and 10 μm, respectively.

In the next step, we evaluated if the characterized MP-p29 interaction could modify the intracellular redistribution of both proteins. For this purpose, first we analyzed the intracellular distribution of each protein separately. To do this, binary constructions containing the CiLV-C p29-eGFP or MP-eGFP fusion proteins were used for transient expression in *N. benthamiana* leaves. The fluorescent signal derived from the p29-eGFP construct accumulated in numerous punctate bodies (Fig. 6A, a, white arrows) and in large inclusions bodies (Fig. 6A, a, red arrows) dispersed throughout the cytoplasm, as reported previously^28^. Callose staining revealed that p29 derived GFP signal was also detected in the 8.3% of PD (Table 1) (Fig. 6A, c, blue arrow; PCC = 0.14). Unlike the p29, MP-eGFP expression accumulated in small punctate structures along the plasma membrane (Fig. 6A, d; PCC = 0.84), which co-localized with the 92.3% of stained PD (Table 1), as reported previously^28^.

In the next step, the subcellular localization of the p29 was evaluated in presence of the MP. To do that, the p29-eGFP construct was co-infiltrated with the MP-HA construct. We observed a clear redistribution of the small punctate p29-eGFP structures from the cytoplasm to the cell periphery, especially along the plasma membrane (Fig. 6B,a), which co-localized correctly (PCC = 0.79) with the PD (Fig. 6B,b, blue arrow), incrementing the percentage of stained PD which co-localize with the p29-GFP derived signal (8.3% to 58.5%; Table 1). Apparently, the presence of the MP redirected the p29 to the PD structures.

Based on the suggested capacity of the MP to interact with the p29 with both N- and C-termini, we investigated the role of both MP regions on the p29 redistribution. First, we characterized the sub-localization of these regions (MPΔ_1–227_ and MPΔ_228–297_) by fusion the eGFP at its C-termini (MPΔ_228–297_-eGFP and MPΔ_1–227_-eGFP). The N-terminal region of the MP (MPΔ_228–297_-eGFP) accumulated in punctate structures at the cell periphery (Fig. 6C,a), similar to that observed for the MP wt, but did not coincide completely (PCC = 0.42) with the PD (see Fig. 5b), reducing the percentage of stained PD showing MPΔ_228–297_-eGFP derived signal, when compared to the MP wt (92.3% vs 45.2%; Table 1). Unlike the N-terminal region, the C-terminal 70 residues of the MP (MPΔ_1–227_-eGFP) resulted in a diffuse GFP signal along the plasma membrane (Fig. 6C,b) which did not co-localize with PD (Fig. 6C,c; PCC =  − 0.04; Table 1).

Finally, BiFC analysis were performed by transiently co-expression of the p29 protein with the N (MPΔ_228–297_) or C (MPΔ_1–227_) MP regions in *N. benthamiana* leaves. The YFP signal revealed that the MP C-terminal region interacts with the p29 forming a complex distributed in the cytoplasm (red arrow in Fig. 6D,a) and along the cell periphery (blue arrow in Fig. 6D,a enlarged), indicating that this portion of the MP is able to recruit the p29 protein from the cytoplasm to the periphery. The MP C-terminal redistribution furthermore indicates the capability of interactions between p29 and this MP fragment. Callose staining revealed that the cell periphery fluorescence derived from the MP_Δ1–227_-p29 complex did not coincide completely with the PD structures (PCC = 0.38) (Fig. 6D,c, blue arrows) allowing its detection in the 28.7% of stained PD (Table 1). On the other hand, we observed that the BiFC signal derived from the MPΔ_228–297_-p29 interaction accumulated in aggregates throughout the cytoplasm (Fig. 6D,b) which do not co-localize with PD (Table 1). No interactions were detected for any of the negative controls analyzed (P29-CYFP + Ncyt, MPΔ_1–227–_CYFP + Ncyt and MPΔ_228–297_-CYFP + Ncyt, see Fig S4g–i). Collectively, these results further indicate that, being both N- and C-terminal regions of CiLV-C MP able to interact with the p29, only the C-terminal portion of the MP is able to direct this complex to the cell periphery.

### The MP of cilevirus accesses the nucleus and interacts with the fibrillarin in the nucleolus

**Figure 7:**
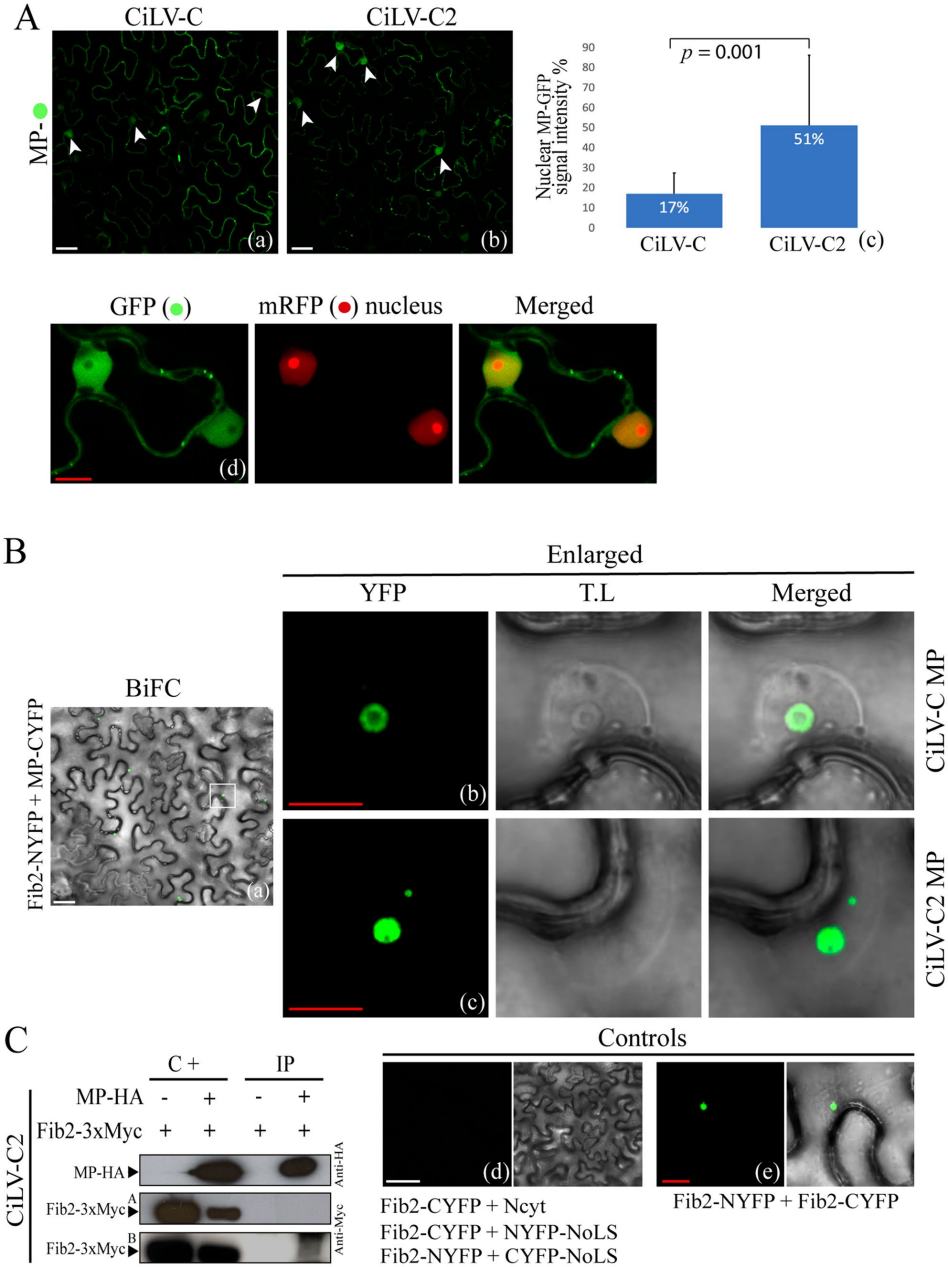
The cileviruses MP interacts with fibrillarin (Fib2) into the nucleolar compartment. (**A**) The CiLV-C MP (a) and CiLV-C2 MP (b) carrying the eGFP (green filled circle) were solely expressed (a,b) and co-expressed (c) with a nucleus marker (red filled circle) in leaf epidermal cells of *N. benthamiana* plants. The white arrows indicate the diffuse MP GFP signal into the nucleus (a,b) which co-localize with the nucleus marker. (c), histogram represents the nuclear GFP intensity observed with the CiLV-C and CiLV-C2 MPs. GFP intensity signals of 20 distinct nuclei for each construct were measured using Image J (version 2.0r) Macros plugin. The nucleus GFP signal is represented in percentage. Student’s *t*-test and significance was set at *p* < 0.05. The *p*-value obtained from comparison between pair of groups is presented. (**B**) BiFC analysis of the interaction between CiLV-C/CiLV-C2 MPs and Fib2. *N. benthamiana* fibrillarin was targeted at its C-terminus with NYFP and CYFP and co-expressed with the MP labelled at its N- or C-terminus with the NYFP or CYFP. Representative protein pair combinations are indicated at the left or bottom of each image. (a) Merged image indicating YFP fluorescence return in several nuclei. Magnification images shows YFP signal into the nucleus, suggesting interaction between MP of CiLV-C (b) or CiLV-C2 (c) and Fib2. Negative control (d) corresponds to the expression of the Fib2 protein in combination with N-Cyt vector and NoLS construct that correspond to a nucleolar peptide signal (RKRHAKKK)^66^ fused at the C-terminus of the YFP fragments. Positive control (e) corresponds to the dimerization of the Fib2 protein by Fib2-NYFP + Fib2-CYFP co-expression. The fluorescent signals were visualized at 72 h post-infiltration and, for the BiFC, at four days post-infiltration. The green (GFP), red (nucleus marker), transmitted light (T.L) channels and merged images are shown in the figure. Withe and red bars correspond to 50 and 10 μm, respectively. The BiFC images displayed are representative of at least three independent experiments. (**C**) Co-immunoprecipitation of CiLV-C2 MP with Fib2. Agrobacteria cultures containing MP-HA and Fib2-3xMyc plasmids, were co-infiltrated into *N. benthamiana* leaves and extracts were analyzed at 3 days post infiltration. HA and Myc antibodies were used in the western blots. A, leaf extract treated with a non-denaturing buffer; B, leaf extract treated with RIPA buffer; C+, positive controls (samples non-immunoprecipitated) and IP, immunoprecipitated samples. The + and − signs indicate the presence or absence of the corresponding proteins in the leaf extracts.

During MP subcellular distribution assay and, unlike the majority of MPs of the 30K family^8,37–40^, we extensively visualized the presence of MP-eGFP signal within the nuclei of *N. benthamiana* epidermal cells (see Fig. 6A,b; the protoplasts in Fig. 4, panels b,d; and Fig. 7A, panels a and b, white arrows), which was confirmed with the co-expression with a nucleus marker fused to the mRFP (mRFP-pCB302) (Fig. 7A,d). This result suggests that the nucleus could has an implication on the movement mechanism mediated by cilevirus MPs. The nuclear localization of CiLV-C2 MP was visually more evident when compared with the CiLV-C MP (Fig. 7A, panel a,b). In this sense, the analysis of the fluorescent signal derived from 20 different nuclei from leaves infiltrated with the same adjusted *Agrobacterium* density (OD_600_ = 0.5) showed that CiLV-C2 MP content was significantly higher into the nucleus (GFP intensity signal of 51.21%) than CiLV-C MP (17.11%) (Fig. 7A,c; *p*-value < 0.05).

Previous studies have reported that nuclear viral MPs are required for the formation of RNPs, in association with CBs and fibrillarin, and that complexes were essential for the viral spread, especially for the systemic movement^41–44^. In this sense, we decided to analyze the putative cilevirus MPs-fibrillarin interaction in the nucleolar compartment, by BiFC assay. To this end, fibrillarin constructs carrying C-terminal fusions of the NYFP or CYFP fragments were co-expressed with the CiLV-C and CiLV-C2 MPs constructs containing the YFP counterpart fused at their N- or C-terminus in *N. benthamiana* leaves. Reconstitution of the fluorescent signal was detected in the nucleolar compartment of several cells (Fig. 7B,a–c), suggesting the capacity of the cileviruses MP to interact with fibrillarin. No interactions were detected for negative controls (Fib2 + Ncyt, Fib2 + NoLS or MP + Ncyt, MP + Cer, MP + TSWV N; Figs. 7c,d, S4g). The controls images are representative of the equivalent interaction observed for both MPs. To confirm this interaction, Co-IP experiments between CiLV-C2 MP and Fib2 were performed. This MP was chosen for Co-IP, since it accumulated in significantly higher levels into the nucleus than CiLV-C MP. Given that the interaction takes place inside the nucleus, two different lysis buffers were used to show the intranuclear location of the MP-Fib2 interaction. The RIPA buffer, containing ionic detergents and active constituents that permit the nuclear membrane disruption^45^, and a non-denaturing lysis buffer, carrying nonionic detergent (NP-40), which does not disrupt the cell nucleus^46^. *N. benthamiana* leaves were infiltrated with agrobacterium cultures carrying the expression cassettes for the CiLV-C2 MP-HA and Fib2-3xMyc constructs and subjected to the Co-IP assay at 3 days post infiltration. Using the non-denaturing lysis buffer, no band was visualized in the MP-HA + Fib2-3xMyc immunoprecipitated extract after western blot analysis (Fig. 7C, western panel A). However, when the same infiltrated leaves were treated with RIPA buffer, a clear band was observed in the immunoprecipitated extract (Fig. 7C, western panel B), indicating both the capacity of the MP to interact with fibrillarin but also that such interaction occurs in the nucleus.

## Discussion

Although cileviruses have been known for decades and reported in more than 50 natural and experimental host species so far^19,47^, nothing is known about their movement machinery. In this work, we reveal in detail the functionality of the p32 protein encoded by CiLV-C and CiLV-C2. Ours findings provide a direct experimental evidence to support the movement function for the p32 protein, which was able to restore cell-to-cell and systemic movement of a MP defective AMV infectious clone. We also tested this ability for all other encoded cilevirus proteins all being unable to restore the AMV transport, resulting in infection of individual cells. Additionally, the CiLV-C p32 was also able to complement *in trans* the movement-deficiency phenotype of a turnip crinkle virus **(**TCV) and tobacco mosaic virus (TMV) mutants in *N. benthamiana* plants^48,49^, indicating the capacity of cilevirus MP to rescue the local movement of viruses that differ in movement transport mechanisms.

In the AMV transport mechanism, the C-terminal A44 residues of AMV MP retain the capacity to interact with the cognate CP, facilitating that heterologous MPs complement the AMV cell-to-cell and systemic movement^9^. This compatibility was previously tested for ilar-, bromo-, cucumo-, como- and orthotospoviruses MPs, which are only functional when carrying the A44 fragment^9,30,31,50^. Here, the MP of cileviruses were competent to complement the cell-to-cell AMV transport regardless of the A44 extension, suggesting that the transport of AMV RNA by this MP is independent of the CP. Additionally, the hypothesis that the movement generated could be the product of a putative interaction between these heterologous MPs with the CP of AMV was excluded from in vivo interaction analysis, further suggesting our inferences above mentioned. In addition, the positive virus transport observed with the AMV RNA3 derivative carrying a CP gene defective in virion assembly (CPN199)^8,33^ clearly shows that the cilevirus MPs are able to mediated the transport of other viral complexes different than the virus particles and independent of the CP, at least in the AMV context. The same ability to complement the AMV transport regardless of the A44 extension, was previously demonstrated for the TMV MP in the AMV context^9^. This protein is able to bind nucleic acids non-specifically, to form RNP complexes in association with viral replication proteins and host factors to traffic the RNPs intracellularly to the cell periphery, and to mediate the passage through the PD without the involvement of CP^12^. Here, we do not observe a significant decrease in movement efficiency comparing the non-fused A44 constructs with the AMV wt. The cileviruses MPs capacity to support the AMV transport without the CP aid strongly suggest that these proteins show a putative high RNA affinity, a feature identified in MPs of various positive stranded-RNA viruses^51^. However, and in spite that the CP was not required for the cilevirus MPs in the AMV context, we cannot exclude the participation of capsid protein in virus transport in the natural CiLV-C infection.

Interestingly, the CiLV-C2 MP was able to complement the systemic movement of AMV non-associated with the A44 fragment, despite this portion of the AMV MP is essential for the systemic movement of AMV^8,33,52^. Based on these findings, we can conclude that this protein seems competent enough to transport, throughout the phloem, infective viral complex regardless of the interaction with the coat protein, indicating that virus particles are not required for the AMV systemic transport, at least with this viral MP. Further experiments will be addressed to know which viral complexes are systemically transported with the CiLV-C2 MP.

Previous studies have suggested that under natural conditions of BTVs infection, the cileviruses and dichorhaviruses are not able to reach their plant hosts systemically^19^. Here, our findings clearly indicate that MPs of cileviruses are competent to allow the systemic transport of a movement defective viral clone in *Nicotiana tabacum* species. In this sense, we speculate that limitation of the cileviruses to reach their hosts systemically in the natural infection process is not due the functional restriction in their MPs, but possibly to some host defense factors. A general mechanism such as RNA silencing is more likely to be responsible for this viral limited movement phenotype^48^. However, further experiments are needed to address this question.

By BiFC analysis, we observed that the nuclear localization of the cilevirus MPs correlated with their capacity to interact with the fibrillarin in the nucleolus compartment. Similar result was observed by Co-IP experiments using the CiLV-C2 MP but only when a RIPA buffer was used which disrupts the nuclear membranes, suggesting that the MP-fibrillarin complex is located mainly in the nuclear extract. The same nuclear localization property was attributed to other viral proteins associated with viral movement. The viral proteins p20 (potexvirus), p2 (tenuivirus), Triple Gene Block 1 (TGB1) (hordeivirus, pomovirus), P7a (betanecrovirus), VPg (potyvirus) and ORF 3 (umbravirus) have been shown to cycle through the nucleus associating with Cajal bodies (for some cases) and fibrillarin, a route required for the formation of RNPs and essential for viral cell-to-cell movement and systemic spread^42,44^. Although speculative, it is tempting to hypothesize that the MP of cileviruses would act similarly favoring the viral movement. In this sense, the higher nuclear localization observed for the CiLV-C2 MP (compared with CiLV-C MP) correlated with a more efficient systemic movement. However, further experiments, using silenced fibrillarin plants, will be addressed to clarify the role of fibrillarin in the viral transport mediated by cilevirus MPs.

Another feature explored in unraveling the performance of the cileviruses MP in viral transport was the ability of MP to induce the formation of tubular structures on the surface of the protoplasts^53^. Sequential deletion in the C-terminal of CiLV-C2 MP showed that, although the absence of this fragment still enables cell-to-cell movement, its removal impairs the correct tubule polymerization (resulting in short tubules) and MP-plasmodesma association, reducing the percentage of PD targeted with cilevirus MPs from 92.3 (wt) to 45.2% (CiLV-C MPΔ_228–297_). Interestingly, the functional cilevirus MPs, lacking the large C-terminal region, showed a reduced cell-to-cell transport and were unable to support the viral systemic transport, which correlated with the reduced MP-plasmodesma association and/or the short tubular structures. However, this correlation showed herein was not reproduced for other viruses. For instance, the AMV MP mutant (Δ242–256) induced tubular structures in less that 5% of the assayed protoplasts, whereas this mutation permitted wilt type levels of foci formation^8^. Other example is the MP of the TSWV, when expressed in TMV system with a deletion of the C-terminal 54 residues rendered a protein unable to generate tubular structures, but still functional with a reduced cell-to-cell transport^54^. For the cilevirus constructs, the absence of tubules or the presence of short- or large tubules resulted in the absence of movement, low efficient movement or highly efficient movement, respectively.

The cilevirus MPs tolerate a C-terminal deletion of -70 or -69 amino acids. Similar C-terminal deletions (60 amino acids or less) did not interfere with the movement functions of the 30 kDa MP of TMV, AMV, cucumber mosaic virus (CMV), cowpea chlorotic mottle virus (CCMV) and odontoglossum ring sport virus (ORSV)^8,34,55,56^. Additional deletions (-72 or -74 amino acids) of the cilevirus MPs inhibited the movement, suggesting that the three or four residues indicated here as essential for the movement (“LIV” for CiLV-C MP and “GMVT” for CiLV-C2 MP, Fig. 3A) are possibly responsible to ensure a correct three-dimensional structure critical for MP polymerization and/or tubule formation. However, we do not exclude that the reduced length of MPs could influence the inhibition of movement. The systemic movement was completely impaired when the C-terminal region was removed (CiLV-CΔ_228–297_ and CiLV-C2Δ_222–292_), despite the fact that these constructions were still functional for a reduced cell-to-cell transport. In this sense, the observation that a minimal cell-to-cell speed is required for the virus to reach the upper region of the plant^31,57^, could justify the systemic infection impediment observed with these truncated MPs.

The MP, when co-expressed with the p29 protein resulted in a redistribution of the p29 from cytoplasm to cell periphery. This observation suggests that the MP coordinates intracellular trafficking of its cognate viral coat protein. More significantly, the C-terminus, but not the N-terminus, of the cileviruses MP proved to be the region responsible to orchestrate the trafficking of the p29 to the cell periphery. Intriguingly, deletion of the MP N-terminus completely abolished its localization with the plasmodesma, but not its distribution at the cell periphery, indicating that the PD localization sequence is located at the N-terminus of the cilevirus MPs, as recently identified in the N-terminus of TMV MP^58^. In addition to the MP, the cilevirus CPs were also able to reach the plasmodesmata structures without any other viral protein, a process that was incremented by the presence of MP through its C-terminus. Similar autonomous PD localization has been described for other viral CP, involving post-translational modifications^59^. Altogether, indicate that the C-terminal region of cilevirus MPs is a critical *cis* element required for both the systemic transport and the incorporation of coat protein in the viral complexes transported through PD. The implication of the C-terminal region of MPs assigned to the 30K family, in viral systemic transport has been previously described^8,36,60^, but also the requirement of the CP, either in form of ribonucleoprotein complex or virus particles, through an interaction to this MP region^9, 31,36,52,61^. The results presented herein indicate that the critical region required for the viral systemic transport is only the C-terminus of cilevirus MPs through its role in tubule formation and PD localization, which contribute to a more efficient cell-to-cell transport. The interaction of this region with the cognate CP could be considered a *cis* element to ensure the transport of the CP required for other viral processes (e.g. vector transmission, RNA protection, etc.) but, apparently, unnecessary for the systemic transport. The open question is if this model could be applied to the rest of MPs assigned to the 30K family.

**Figure 8:**
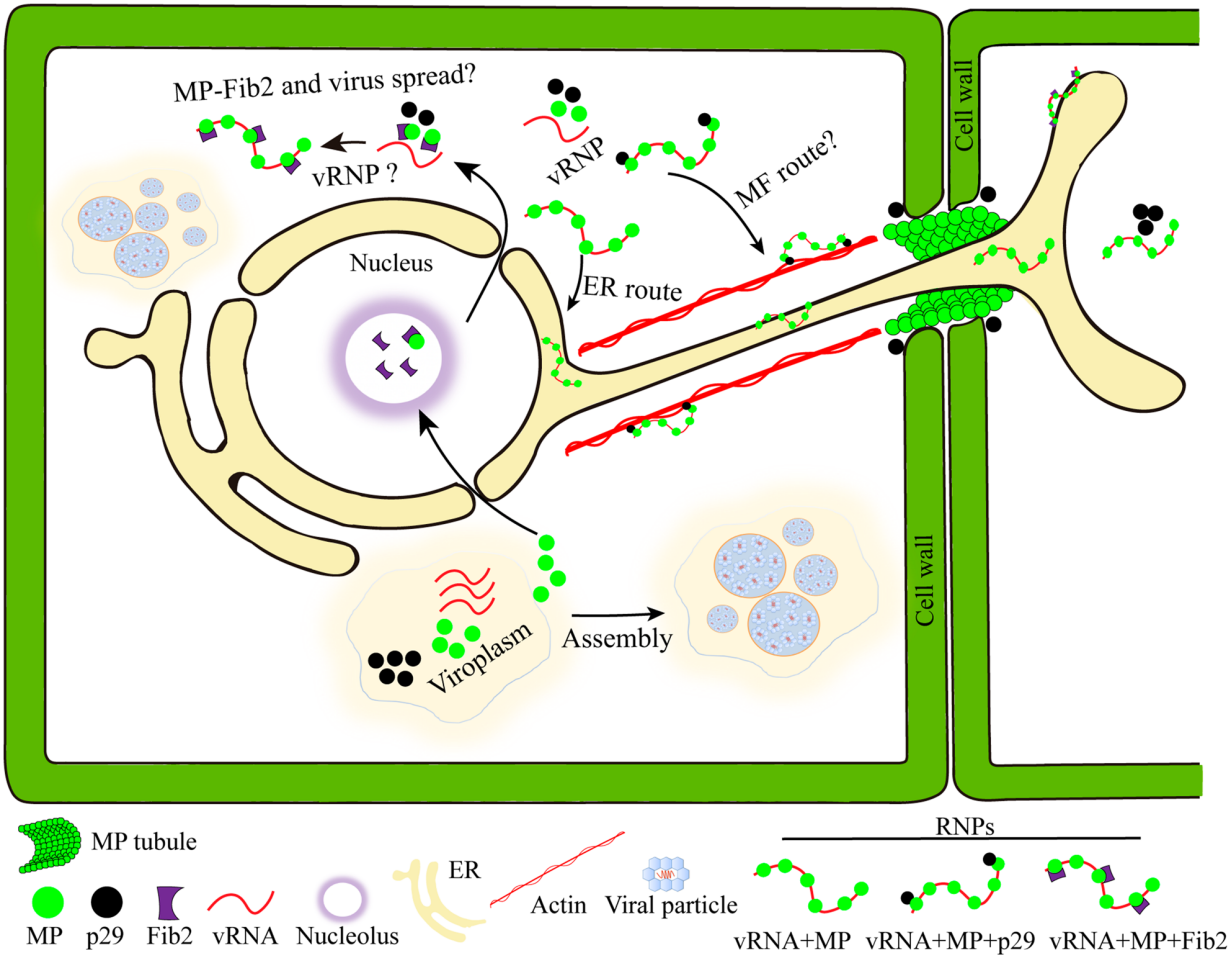
Model for the role of the cileviruses MP in viral intracellular and intercellular transport. The cileviruses replication occurs in the membranes of endoplasmic reticulum (ER) resulting in larger viroplasms. A portion of the MP is transported into the nucleolus of the cell to bind to fibrillarin. The possibility to form a complex between MP plus fibrillarin that could exit the nucleus and interacts with vRNA and/or CP (p29) to form infectious vRNPs, is an open question. The MP, a membrane spanning protein, may anchors the vRNP complex to the ER membrane network, which traffics by the ER system to neighboring cells, facilitating the passage through plasmodesmata by the tubule formation. A possible alternative route may be mediated by the capacity of p29 to interact with MP and to associate with actin, thus anchoring the infectious complexes along the microfilaments (MF), guiding the vRNPs throughout the cytoplasm to the cell periphery. The virus particle is not required for the intercellular transport for this MP (indicating its capability to transport viral complexes different to virus particles intra and intercellularly); furthermore, the MP can transport the infectious complex cell-to-cell and systemically independent of the CP assistance. The redirection of p29 by MP to the plasmodesma could also be implicated to initiate viral replication in the newly infected cells.

Cileviruses accumulate and replicate in the cytoplasm, remodeling the membrane of the ER network and generating large viroplasms^28,62,63^. Our previous study has shown that MP and p29 traffic along the ER system, and the MP is integrally associated with the cell membrane^28^. Taken together with the data showed herein, these findings support a model for the role of the cileviruses MP in viral spread (Fig. 8). Our model posits that the MP is translated in the viroplasm and a portion of its population is transported into the nucleolus of the cell to bind to fibrillarin. This points to a scenario in which the MP could manipulate or recruit nucleolar functions to promote the movement, however whether the core complex formed is exported from the nucleus to the cytoplasm to form infective vRNPs and its association with viral movement, requires additional investigation. The p32, a membrane spanning protein, may anchor the vRNP complex to the ER membrane network, which traffics by the ER system to neighbor cells regardless the CP assistance. The observation that p29 has the capacity to interact with the MP and to traffic along the ER, and probably through the actin system^28^, opens an alternative route, in which the RNP complexes, carrying both p29 and MP proteins, could be transported to the cell periphery along the actin filaments. Finally, a part of the MP population is recruited to PD for tubule formation, which facilities the passage of the infective complexes throughout the PD to neighbor cells, a step in which the MP is able to generate independently of viral particle assembly, indicating its capability to transport viral complexes different to virus particles intra and intercellularly. However, the redirection of p29 to the plasmodesma through its interaction with the MP could also be implicated in the initiation of viral replication in the newly infected cells. This model represents a start point for unraveling the movement mechanism of the cileviruses.

## Materials and methods

### DNA manipulation

Details on plasmid construction are presented in Supplementary Materials and Methods.

### Organelle markers

For the nucleus subcellular co-localization, the proteins were co-infiltrated with cultures (OD_600_ = 0.1) expressing the NLS of SV40 large T antigen (nucleus marker) fused to the red fluorescent protein (λexc = 587 nm; λem = 610 nm), provided by Dr. José Navarro, IBMCP, Valencia, Spain.

### Protoplast preparation and inoculation of P12 plants

All details on protoplast preparation and inoculation of P12 plants are presented in Supplementary Materials and Methodss.

### Tissue-printing assay

Tissue-printing analysis were performed by transversal sections of the corresponding petiole from inoculated (I) and upper (U) P12 leaves at 14 dpi, as performed previously^64^. All results showed from tissue-printing are representatives from three independent assays. The nucleic acids were fixed to the nylon membranes using UV cross-linker (700 × 100 μJ/cm^2^). Hybridization and detection were conducted as described^65^, using a Dig-riboprobe (Roche Mannheim, Germany) complementary to the AMV 3′ UTR.

### Intracellular sublocalization and BiFC assay

All details on protein expression for sublocalization and in vivo protein–protein interaction are presented in Supplementary Materials and Methods.

### Confocal laser scanning microscopy

Fluorescence images were captured with the aid of a confocal laser scanning microscope Zeiss LSM 780 model. The excitation and emission captures of the GFP, YFP, mRFP fluorophores and aniline blue fluorochrome were conducted as described^28^. The images were prepared using Fiji Image J program (version 2.0r). For a better interpretation of the co-localization, graphs of fluorescence intensity were generated with Zeiss quantify intensity tools software. GFP intensity signals of 20 distinct nuclei were measured using Image J (version 2.0r) Macros plugin, the calculation area was selected to have GFP signal of similar intensities.

### Co-IP assays

All details for in vivo protein–protein interactions are presented in Supplementary Materials and Methods.

### Ethical approval

This article does not contain any studies with human participants or animals requiring ethical approval.

## Supplementary Information

Supplementary Information.

## References

[1] Morozov SY, Solovyev AG (2003). Triple gene block: Modular design of a multifunctional machine for plant virus movement. J. Gen. Virol..

[2] Lazareva EA (2017). A novel block of plant virus movement genes. Mol. Plant Pathol..

[3] Lucas WJ (2006). Plant viral movement proteins: Agents for cell-to-cell trafficking of viral genomes. Virology.

[4] Navarro JA, Sanchez-Navarro JA, Pallas V (2019). Key checkpoints in the movement of plant viruses through the host. Adv. Virus Res..

[5] Hofmann C, Niehl A, Sambade A, Steinmetz A, Heinlein M (2009). Inhibition of tobacco mosaic virus movement by expression of an actin-binding protein. Plant Physiol..

[6] Kawakami S, Watanabe Y, Beachy RN (2004). Tobacco mosaic virus infection spreads cell to cell as intact replication complexes. Proc. Natl. Acad. Sci. U.S.A..

[7] Sambade A, Heinlein M (2009). Approaching the cellular mechanism that supports the intercellular spread of Tobacco mosaic virus. Plant Signal. Behav..

[8] Sanchez-Navarro JA, Bol JF (2001). Role of the alfalfa mosaic virus movement protein and coat protein in virus transport. Mol. Plant Microbe Interact..

[9] Sanchez-Navarro JA, Carmen Herranz M, Pallas V (2006). Cell-to-cell movement of Alfalfa mosaic virus can be mediated by the movement proteins of Ilar-, bromo-, cucumo-, tobamo- and comoviruses and does not require virion formation. Virology.

[10] Ritzenthaler C, Hofmann C, Waigmann E, Heinlein M (2007). Tubule-guided movement of plant viruses. Viral Transport in Plants.

[11] Benitez-Alfonso Y, Faulkner C, Ritzenthaler C, Maule AJ (2010). Plasmodesmata: Gateways to local and systemic virus infection. Mol. Plant Microbe Interact..

[12] Niehl A, Heinlein M (2011). Cellular pathways for viral transport through plasmodesmata. Protoplasma.

[13] Scholthof HB (2005). Plant virus transport: Motions of functional equivalence. Trends Plant Sci..

[14] Heinlein M, Epel BL (2004). Macromolecular transport and signaling through plasmodesmata. Int. Rev. Cytol..

[15] Wolf S, Deom CM, Beachy RN, Lucas WJ (1989). Movement protein of tobacco mosaic virus modifies plasmodesmatal size exclusion limit. Science.

[16] Kaplan IB, Zhang L, Palukaitis P (1998). Characterization of cucumber mosaic virus. V. Cell-to-cell movement requires capsid protein but not virions. Virology.

[17] Caranta C, Aranda MA, Tepfer M, Lopez-Moya JJ (2011). Recent Advances in Plant Virology.

[18] Hipper C, Brault V, Ziegler-Graff V, Revers F (2013). Viral and cellular factors involved in Phloem transport of plant viruses. Front. Plant Sci..

[19] Freitas-Astua J, Ramos-Gonzalez PL, Arena GD, Tassi AD, Kitajima EW (2018). Brevipalpus-transmitted viruses: Parallelism beyond a common vector or convergent evolution of distantly related pathogens?. Curr. Opin. Virol..

[20] Dietzgen RG (2018). Dichorhaviruses in their host plants and mite vectors. Adv. Virus Res..

[21] Bastianel M, Novelli V, Kitajima E, Kubo K, Bassanezi RB (2010). Citrus leprosis: Centennial of an unusual mite virus pathosystem. Plant Dis..

[22] Roy A (2013). A novel virus of the genus Cilevirus causing symptoms similar to citrus leprosis. Phytopathology.

[23] Roy A (2015). Role bending: Complex relationships between viruses, hosts, and vectors related to citrus leprosis, an emerging disease. Phytopathology.

[24] Leon MG, Becerra CH, Freitas-Astua J, Salaroli RB, Kitajima EW (2008). Natural infection of *Swinglea glutinosa* by the citrus leprosis virus cytoplasmic type (CiLV-C) in Colombia. Plant Dis..

[25] Pascon RC (2006). The complete nucleotide sequence and genomic organization of citrus leprosis associated virus, cytoplasmatic type (CiLV-C). Virus Genes.

[26] Locali-Fabris EC (2006). Complete nucleotide sequence, genomic organization and phylogenetic analysis of citrus leprosis virus cytoplasmic type. J. Gen. Virol..

[27] Mushegian AR, Elena SF (2015). Evolution of plant virus movement proteins from the 30K superfamily and of their homologs integrated in plant genomes. Virology.

[28] Leastro MO, Kitajima EW, Silva MS, Resende RO, Freitas-Astua J (2018). Dissecting the subcellular localization, intracellular trafficking, interactions, membrane association, and topology of citrus leprosis virus C proteins. Front. Plant Sci..

[29] Sanchez-Navarro J, Miglino R, Ragozzino A, Bol JF (2001). Engineering of alfalfa mosaic virus RNA 3 into an expression vector. Arch. Virol..

[30] Leastro MO, Pallas V, Resende RO, Sanchez-Navarro JA (2017). The functional analysis of distinct tospovirus movement proteins (NSM) reveals different capabilities in tubule formation, cell-to-cell and systemic virus movement among the tospovirus species. Virus Res..

[31] Fajardo TV, Peiro A, Pallas V, Sanchez-Navarro J (2013). Systemic transport of Alfalfa mosaic virus can be mediated by the movement proteins of several viruses assigned to five genera of the 30K family. J. Gen. Virol..

[32] Mas P, Pallas V (1995). Non-isotopic tissue-printing hybridization: A new technique to study long-distance plant virus movement. J. Virol. Methods.

[33] Tenllado F, Bol JF (2000). Genetic dissection of the multiple functions of alfalfa mosaic virus coat protein in viral RNA replication, encapsidation, and movement. Virology.

[34] Nagano H, Okuno T, Mise K, Furusawa I (1997). Deletion of the C-terminal 33 amino acids of cucumber mosaic virus movement protein enables a chimeric brome mosaic virus to move from cell to cell. J. Virol..

[35] Aparicio F, Pallas V, Sanchez-Navarro J (2010). Implication of the C terminus of the *Prunus necrotic* ringspot virus movement protein in cell-to-cell transport and in its interaction with the coat protein. J. Gen. Virol..

[36] Takeda A, Kaido M, Okuno T, Mise K (2004). The C terminus of the movement protein of Brome mosaic virus controls the requirement for coat protein in cell-to-cell movement and plays a role in long-distance movement. J. Gen. Virol..

[37] Herranz MC, Sanchez-Navarro JA, Aparicio F, Pallas V (2005). Simultaneous detection of six stone fruit viruses by non-isotopic molecular hybridization using a unique riboprobe or 'polyprobe'. J. Virol. Methods.

[38] Canto T, Palukaitis P (2005). Subcellular distribution of mutant movement proteins of cucumber mosaic virus fused to green fluorescent proteins. J. Gen. Virol..

[39] Margaria P, Anderson CT, Turina M, Rosa C (2016). Identification of Ourmiavirus 30K movement protein amino acid residues involved in symptomatology, viral movement, subcellular localization and tubule formation. Mol. Plant Pathol..

[40] Heinlein M (1998). Changing patterns of localization of the tobacco mosaic virus movement protein and replicase to the endoplasmic reticulum and microtubules during infection. Plant Cell.

[41] Zheng L (2015). Rice stripe tenuivirus p2 may recruit or manipulate nucleolar functions through an interaction with fibrillarin to promote virus systemic movement. Mol. Plant Pathol..

[42] Li Z (2018). Hijacking of the nucleolar protein fibrillarin by TGB1 is required for cell-to-cell movement of Barley stripe mosaic virus. Mol. Plant Pathol..

[43] Taliansky ME, Brown JW, Rajamaki ML, Valkonen JP, Kalinina NO (2010). Involvement of the plant nucleolus in virus and viroid infections: Parallels with animal pathosystems. Adv. Virus Res..

[44] Kim SH (2007). Interaction of a plant virus-encoded protein with the major nucleolar protein fibrillarin is required for systemic virus infection. Proc. Natl. Acad. Sci. U.S.A..

[45] Alcaraz C, De Diego M, Pastor MJ, Escribano JM (1990). Comparison of a radioimmunoprecipitation assay to immunoblotting and ELISA for detection of antibody to African swine fever virus. J. Vet. Diagn. Investig..

[46] Rosner M, Hengstschlager M (2008). Cytoplasmic and nuclear distribution of the protein complexes mTORC1 and mTORC2: Rapamycin triggers dephosphorylation and delocalization of the mTORC2 components rictor and sin1. Hum. Mol. Genet..

[47] Garita LC, Tassi AD, Calegario RF, Freitas-Astúa J (2014). Experimental host range of citrus leprosis virus C (CiLV-C). Trop. Plant Pathol..

[48] Leastro MO (2020). Citrus leprosis virus C encodes three proteins with gene silencing suppression activity. Front. Microbiol..

[49] Leastro MO, Freitas-Astua J, Kitajima EW, Pallas V, Sanchez-Navarro JA (2020). Dichorhaviruses movement protein and nucleoprotein form a protein complex that may be required for virus spread and interacts in vivo with viral movement-related cilevirus proteins. Front. Microbiol..

[50] Peiro A (2014). The movement protein (NSm) of Tomato spotted wilt virus is the avirulence determinant in the tomato Sw-5 gene-based resistance. Mol. Plant Pathol..

[51] Taliansky M, Torrance L, Kalinina NO (2008). Role of plant virus movement proteins. Methods Mol. Biol..

[52] Herranz MC, Pallas V, Aparicio F (2012). Multifunctional roles for the N-terminal basic motif of Alfalfa mosaic virus coat protein: Nucleolar/cytoplasmic shuttling, modulation of RNA-binding activity, and virion formation. Mol. Plant Microbe Interact..

[53] Melcher U (2000). The '30K' superfamily of viral movement proteins. J. Gen. Virol..

[54] Lewandowski DJ, Adkins S (2005). The tubule-forming NSm protein from Tomato spotted wilt virus complements cell-to-cell and long-distance movement of Tobacco mosaic virus hybrids. Virology.

[55] Osman F, Schmitz I, Rao AL (1999). Effect of C-terminal deletions in the movement protein of cowpea chlorotic mottle virus on cell-to-cell and long-distance movement. J. Gen. Virol..

[56] Kaplan IB (1995). Complementation of virus movement in transgenic tobacco expressing the cucumber mosaic virus 3a gene. Virology.

[57] Deom CM, He XZ, Beachy RN, Weissinger AK (1994). Influence of heterologous tobamovirus movement protein and chimeric-movement protein genes on cell-to-cell and long-distance movement. Virology.

[58] Yuan C, Lazarowitz SG, Citovsky V (2016). Identification of a functional plasmodesmal localization signal in a plant viral cell-to-cell-movement protein. mBio.

[59] Hung CJ (2014). Phosphorylation of coat protein by protein kinase CK2 regulates cell-to-cell movement of Bamboo mosaic virus through modulating RNA binding. Mol. Plant Microbe Interact..

[60] Kim SH (2004). The C-terminal 33 amino acids of the cucumber mosaic virus 3a protein affect virus movement, RNA binding and inhibition of infection and translation. J. Gen. Virol..

[61] Okinaka Y, Mise K, Suzuki E, Okuno T, Furusawa I (2001). The C terminus of brome mosaic virus coat protein controls viral cell-to-cell and long-distance movement. J. Virol..

[62] Kitajima EW, Chagas CM, Rodrigues JC (2003). Brevipalpus-transmitted plant virus and virus-like diseases: Cytopathology and some recent cases. Exp. Appl. Acarol..

[63] Kitajima EW, Rosillo MA, Portillo MM, Müller GW, Costa AS (1974). Microscopia eletrônica de tecidos foliares de laranjeiras infectadas pela lepra explosiva da Argentina. Fitopatol. Bras. Bras..

[64] Sanchez-Navarro J, Fajardo T, Zicca S, Pallas V, Stavolone L (2010). Caulimoviridae tubule-guided transport is dictated by movement protein properties. J. Virol..

[65] Pallas V, Mas P, Sanchez-Navarro JA (1998). Detection of plant RNA viruses by nonisotopic dot-blot hybridization. Methods Mol. Biol..

[66] Gomez G, Pallas V (2007). A peptide derived from a single-modified viroid-RNA can be used as an "in vivo" nucleolar marker. J. Virol. Methods.

