# Supplementary material for: Unravelling the involvement of cilevirus p32 protein in the viral transport
Source: Sci Rep. 2021 Feb 3;11:2943. doi: 10.1038/s41598-021-82453-4 (PMC7859179; doi:10.1038/s41598-021-82453-4)
Supplement: Supplementary file 1 — Supplementary Information. [file 41598_2021_82453_MOESM1_ESM.docx]

**SUPPLEMENTARY FILES:**

**Unravelling the involvement of cilevirus p32 protein on the viral transport**

Mikhail Oliveira Leastro^1,4^*; Juliana Freitas-Astúa^1,2^, Elliot Watanabe Kitajima^3^, Vicente Pallás^4^ and Jesús A. Sánchez-Navarro^4^*.

^1^Unidade Laboratorial de Referência em Biologia Molecular Aplicada, Instituto Biológico, SP, Brazil;

^2^ Embrapa Mandioca e Fruticultura, Cruz das Almas, BA, Brazil;

^3^Departamento de Fitopatologia e Nematologia, Escola Superior de Agricultura Luiz de Queiroz, Universidade de São Paulo, Piracicaba, SP, Brazil;

^4^Instituto de Biología Molecular y Celular de Plantas, Universidad Politécnica de Valencia-Consejo Superior de Investigaciones Científicas (CSIC), Valencia, Spain.

*Corresponding authors: jesanche@ibmcp.upv.es and m.leastro@gmail.com

**SUPPLEMENTARY MATERIALS AND METHODS**

**DNA manipulation**

*Constructions for studies of cell-to-cell and systemic spread.*

For the analysis of cell-to-cell spread, a modified infectious AMV cDNA 3 clone (pGFP/A255/CP) ^1^, which expresses the green fluorescent protein (GFP), was used to exchange the 255 amino acids (aa) of the AMV *MP* gene with the corresponding putative *MP* of CiLV-C (DQ352194.1) and CiLV-C2 (JX000024). Additionally, all genes (except for the polymerase) from encoded proteins *p29*, *p15*, *p61* and *p24* of CiLV-C were also cloned into the chimeric cDNA 3 of AMV in order to evaluate if other cilevirus proteins have any role on aspects of viral movement. All these genes were amplified from total RNA extracted from infected citrus leaves and fruits with VWR Life Science RiboZol reagent, following the manufacturer’s instructions. The correspondent viral genes were generated from One-Step RT-PCR system (Thermo Fisher Scientific, USA), following the manufacturer’s specification, with specific primers containing the restriction sites *Nco*I, *Pci*I, *BspH*I and *Nhe*I compatible with the AMV system (Fig. 1), to generate the respective constructs: pGFP/*MP*_CiLV-C_:A44/CP, pGFP/*MP*_CiLV-C2_:A44/CP, pGFP/*p29*_CiLV-C_:A44/CP, pGFP/*p15*_CiLV-C_:A44/CP, pGFP/*p61*_CiLV-C_:A44/CP and pGFP/*p24*_CiLV-C_:A44/CP. The resultant heterologous proteins are fused with the C-terminal 44 aa (A44) of the AMV MP, which favors a better efficiency in movement, given that this region is responsible to interact with the cognate AMV capsid protein (CP) ^2^. Primers with stop codon were used to generate constructs without gene fusion with the C-terminus A44 for the CiLV-C and CiLV-C2 MPs, resulting in the construction pGFP/*MP*_CiLV-C_/CP and pGFP/*MP*_CiLV-C2_/CP, respectively.

For the analysis of systemic spread, the *MP* genes aforementioned were introduced in a chimeric infectious cDNA 3 clone of AMV, lacking the GFP gene pAL3NcoP3 ^3^, to generate the constructs p*MP*_CiLV-C_:A44/CP, p*MP*_CiLV-C_/CP, p*MP*_CiLV-C2_:A44/CP and p*MP*_CiLV-C2_/CP. For cileviruses MPs the constructions non-fused to the A44 fragment were also generated.

The pGFP/MP:A44/CP constructs were modified to introduce the *CP* gene lacking the C-terminal 20 amino acids (CPN199), which is defective in virion assembly ^4^. The introduction of the *CP199* gene mutant was performed by exchanging the *Nhe*I-*Pst*I fragment obtained from mutants pGFP/BMV:A44/CP-199 ^5^, to generate the constructs pGFP/AMV/CP-199, pGFP/MP_CiLV-C_:A44/CP-199, pGFP/MP_CiLV-C2_:A44/CP-199 and pGFP/NSm_TSWV_:A44/CP-199.

To evaluate the functional analysis of the C-terminal MP mutants, the truncated CiLV-C and CiLV-C2 *MP* genes (containing or not stop codon) were amplified with restriction sites as above mentioned and introduced in the AMV chimeric constructs pGFP/A255/CP and pAL3NcoP3 for analysis of cell-to-cell and systemic movement, respectively.

*Constructs for tubule formation and subcellular localization*

To evaluate the property of MPs in generate tubular structures, the *MP* genes of CiLV-C and CiLV-C2 were fused at their C-termini, to the eGFP (enhanced Green Fluorescent Protein). Similar C-terminal eGFP fusion was generated with the p29 protein, to assess their subcellular localization. The MPs of cileviruses were HA-tagged at their C-termini for re-localization analysis. The genes containing the restrict sites of *Nco*I, *Pci*I, *BspH*I and *Nhe*I were cloned into the vector pSK35S/GFP:eGFP-PoPit ^6^ under the control of 35S constitutive promoter from cauliflower mosaic virus (CaMV) and the terminator of potato proteinase inhibitor (PoPit). Then, the correspondent expression cassettes were subcloned into the pMOG_800_ binary vector. The C-terminal truncated version of cileviruses MPs: CiLV-CΔ_228-297_, CiLV-CΔ_225-297,_ CiLV-C2Δ_222-292_ and CiLV-CΔ_218-297_ were also cloned in this system as aforementioned.

*Constructions for Bimolecular Fluorescence Complementation (BiFC)*

We performed the BiFC assay with the constructions pSK35S-NYFP:eGFP-PoPit, pSK35S-CYFP:eGFP-PoPit, pSK35S-eGFP:NYFP-PoPit, and pSK35S-eGFP:CYFP-PoPit, which permitted N-and C-terminal fusion of the Enhanced Yellow Fluorescent Protein (EYFP) fragments at the N- or C-terminal of a specific assayed protein by exchange of the eGFP gene using the *Nco*I/*Nhe*I restriction sites. Detailed handling for obtaining these plasmids was previously described ^7^. For the analysis of *in vivo* interaction between the cileviruses MP with the cognate capsid protein (p29), we fused the N-terminal 154 amino acids of the yellow fluorescent protein sequence (NYFP) to the C-terminus of cilevirus MPs wild type (not truncated) and truncated N- and C-terminal versions (CiLV-C_wt_, CiLV-CΔ_1-227_, CiLV-CΔ_228-297_, CiLV-C2_wt_, CiLV-C2Δ_1-221_ and CiLV-C2Δ_222-292_). The C-terminal 84 amino acids of the YFP (CYFP) was fused to the C-terminus of the p29 protein of CiLV-C and CiLV-C2. The expression cassettes containing the MP genes were subcloned into the pMOG_800_ binary vector. The correspondent protein pair combination with N- and CYFP fragment fused to the C-terminal of the MP and p29 proteins was chosen from all possible combinations, previously tested for representing the best visual expression of the EYFP, previously confirmed by ^8^.

The constructions, which contained the N- and C-terminal fragments of the EYFP addressed to the cytosol (NYFPcyt and CYFPcyt) or ER (NYFPer and CYFPer) used here for BiFC control were provided by Dr. Jari P.T Valkonen, University Helsinki ^9^ and by Dr. Frederic Aparicio; Instituto de Biología Molecular y Celular de Plantas “Pirmo Yúfera” (IBMCP), Valencia/Spain ^10^, respectively. The first one-cassette constructions (NYFPer and CYFPer) were obtained from pRT-YN-ER and pRT-YC-ER vectors ^9^ and subcloned into the binary vector pMOG_800_. The BiFC binary plasmids AtFib2-NYFP and AtFib2-CYFP and the negative control NoLS construct, corresponding to a nucleolar peptide signal (RKRHAKKK)^11^ fused at the C-terminus of the YFP fragments, used to evaluate interaction with fibrillarin were gently provided by Msc. Joan Marquez, IBMCP, Valencia/Spain. The BiFC plasmid AMV CP-NYFP and AMV CP-CYFP for cileviruses MP interaction with the AMVC CP was provided by Dr. Frederic Aparicio, IBMCP, Valencia/Spain. The BiFC plasmids TSWV N used as negative control were obtained from ^7^. All DNA manipulations were confirmed by plasmid DNA sequencing.

*Constructions for Co-Immunoprecipitation assays*

For *in vivo* protein-protein interaction by Co-immunoprecipitation assays, the p29, MP wt or its truncated versions and fibrillarin2 (AM269909.1) were fused at their C-termini with the HA (p29 and MP wt) or Myc (all proteins except for the p29) epitopes. All genes were amplified using specific primers where the antisense primers contained the corresponding epitope sequence. The PCR products were introduced in the construct pSK35S-eGFP-PoPit by exchange the eGFP gene using the *Nco*I/*Nhe*I restriction sites. Then, the correspondent expression cassettes were subcloned into the pMOG_800_ binary vector.

**Protoplast preparation and inoculation of P12 plants**

For analysis of tubule polymerization, *A. tumefaciens* (strain C58) cultures (OD_600_ = 0.5) transformed with the corresponding binary vector pMOG_800_ containing the MPs wt and truncated versions above mentioned were used to infiltrate *Nicotiana benthamiana* plants, as previously described ^12^. Three infiltrated leaves per construct were used for protoplasts isolation as described ^13^.

For analysis of cell-to-cell and systemic movements, the cassette from plasmids containing all proteins assayed inserted into AMV 3 cDNA, was amplified with specific

pair primers and the generated amplicons were used directly as template for *in vitr*o transcription with T7 RNA polymerase (Takara Bio Inc). Transgenic *N. tabacum* P12 plants that express the polymerase proteins P1 and P2 of AMV ^14^ were grown and inoculated with RNA transcripts, as previously described ^15^.

Each image-frame expressing GFP represents the visualization of several protoplasts (about 15 to 20) per assay for each MP protein analyzed. GFP expression in protoplast was analyzed with a Zeiss LSM 780 confocal laser-scanning microscope.

The foci images in P12 plants were taken with the aid of a Lupe microscope (MZZ16F Leica) with the area of infection foci measured at two and three days post-inoculation (dpi), using image J software version 2.0r. The graphs represent the average of the area in mm^2^ of 80 independent infection foci from each construct, which error bars indicate the standard deviation. Student’s *t*-test was performed to determine the significant differences between MPs experiments of cell-to-cell efficiency. Significance values with *p* < 0.05 or no significant difference were displayed in the graphics. Each construct was inoculated on three plants with three leaves per plant and used 15 µl of the transcription mixture per leaf as inoculum.

**Intracellular sublocalization and BiFC assay**

To visualize the intracellular distributions of cileviruses MPs and p29 proteins in plant cells, *A. tumefaciens* (strain C58) containing the viral genes fused at their C-terminus with the eGFP or HA tag in pMOG_800_ binary vector, was infiltrated in *N. benthamiana* leaves (OD_600_ = 0.4) as described previously ^7^.

To investigate the co-localization of the MP protein with the nucleus or the redistribution of p29 by interaction with MP, simultaneous expression of two proteins in individual bacteria cultures containing the correspondent binary vectors was performed. For cultures of *A. tumefaciens* transformed with organelle markers in binary vector, OD_600_ was adjusted to 0.5 and mixed with the respective agrobacteria containing the cilevirus genes, before leaf infiltration. The fluorescence was observed at 72h post-infiltration.

For callose staining, *N. benthamiana* leaves were infiltrated with aniline blue (Merk, Darmstadt, Germany) solution at 0.005% concentration in sodium phosphate buffer, 70 mM, pH 9.0. The leaves were infiltrated and kept in a dark room for 2 hours before confocal visualization. Standard deviation in Pearson coefficient correlation (PCC) was measured using the Fiji co-localization plugin for three independent images from approximately 100 individual plasmodesmata. PCC values of 0.2 - 0.4 indicate weak positive correlations, values above 0.5 indicate strong positive correlations ^8,16^.

In BiFC assays, *A. tumefaciens* (strain C58) cultures (OD_600_ = 0.4) transformed with the corresponding binary plasmid pMOG_800_ were used to infiltrate *N. benthamiana* plants as previously described ^12^. To increase the expression, in order to allow a better visualization of the fluorescence signal, all protein pair combinations were co-expressed with the silencing suppressor HCPro from tobacco etch virus. At four days post-infiltration, the fluorescence reconstitution was observed.

For *in vivo* protein-protein interaction, the p29, MP wt and truncated versions of the protein were fused at their N- and C- termini with NYFP and CYFP. The AMV CP was fused at its N-termini with NYFP and CYFP. In heterologous interactions between p29-MP and cileviruses MP-AMV CP the indicated pair of proteins was transiently expressed in *N. benthamiana* as described above.

For BiFC assay addressed to indicate the MP and fibrillarin interaction, the MP carrying the NYFP or CYFP fused at the N- or C-terminus were transiently expressed with the counterpart fused C-terminus with the fibrillarin (Fib2-Nt and Fib2-Ct).

The plants were maintained in FITOTRON plant growth chamber under conditions of 23°C day 18°C night, 70% humidity and 16h light / 8h dark regime. All confocal images of subcellular localization from ectopic protein expression were obtained after repeated visualization of different cells and regions of the *N. benthamiana* leaves. Thus, three independent experiments were performed, each one included the infiltration of three leaves per construct.

**Co-Immunoprecipitation assay (Co-IP)**

The Co-IP assay was addressed using the HA/Myc epitopes and the Pierce Magnetic HA-Tag kit (Thermo Scientific, USA) following the manufacturer instructions. Briefly, *N. benthamiana* plants were agroinfiltrated with the corresponding *A. tumefaciens* cultures carrying the desired expression cassettes, as described above for the BiFC assay. At 3 days post infiltration, 0.1 g of tissue was homogenized with liquid nitrogen and 0.5 ml of non-denaturing buffer (25 mM Tris HCl pH 7.4, 150 mM NaCl, 1 mM EDTA, 1% NP-40, 5% glycerol). For the Fibrillarin-MP interactions, the samples were also homogenized with 0.5 ml of a more denaturing RIPA buffer (50 mM Tris HCl pH 8.0, 150 mM NaCl, 1% Triton X-100, 0.5% sodium deoxycholate, 0.1% SDS). The extracts were incubated on ice for 5 minutes (non-denaturing buffer) or by constant agitation for 2 hours at 4 ºC (RIPA buffer) and then centrifugated at 13,000 x *g* for 10 minutes to remove cell debris. 40 µl of the supernatant were used for the protein expression control (C+) meanwhile the rest of supernatant was incubated with 20 µl (0.2 mg) of Pierce Anti-HA Magnetic Beads during 90 minutes agitation at room temperature. After the washing steps with the non-denaturing buffer, the HA immunoprecipitated proteins were eluted by resuspending the beads with 75 µl of 1X Laemmli buffer and incubate them at 100 ºC for 10 minutes. Western blot analysis were performed using 20 µl of C+ and 30 µl of IP proteins per line and two monoclonal anti-HA and anti-c-Myc antibodies (Sigma-Aldrich, Steinheim, Germany), following the manufactures instructions. For aesthetic reasons, the tissue print and Co-IP membranes are cut in the main figure. Full image of the blunt membranes is demonstrated in supplementary information figures 3 D, 7 C and S4 B.

**SUPPLEMENTARY FIGURES (figs. S1 to S4)**


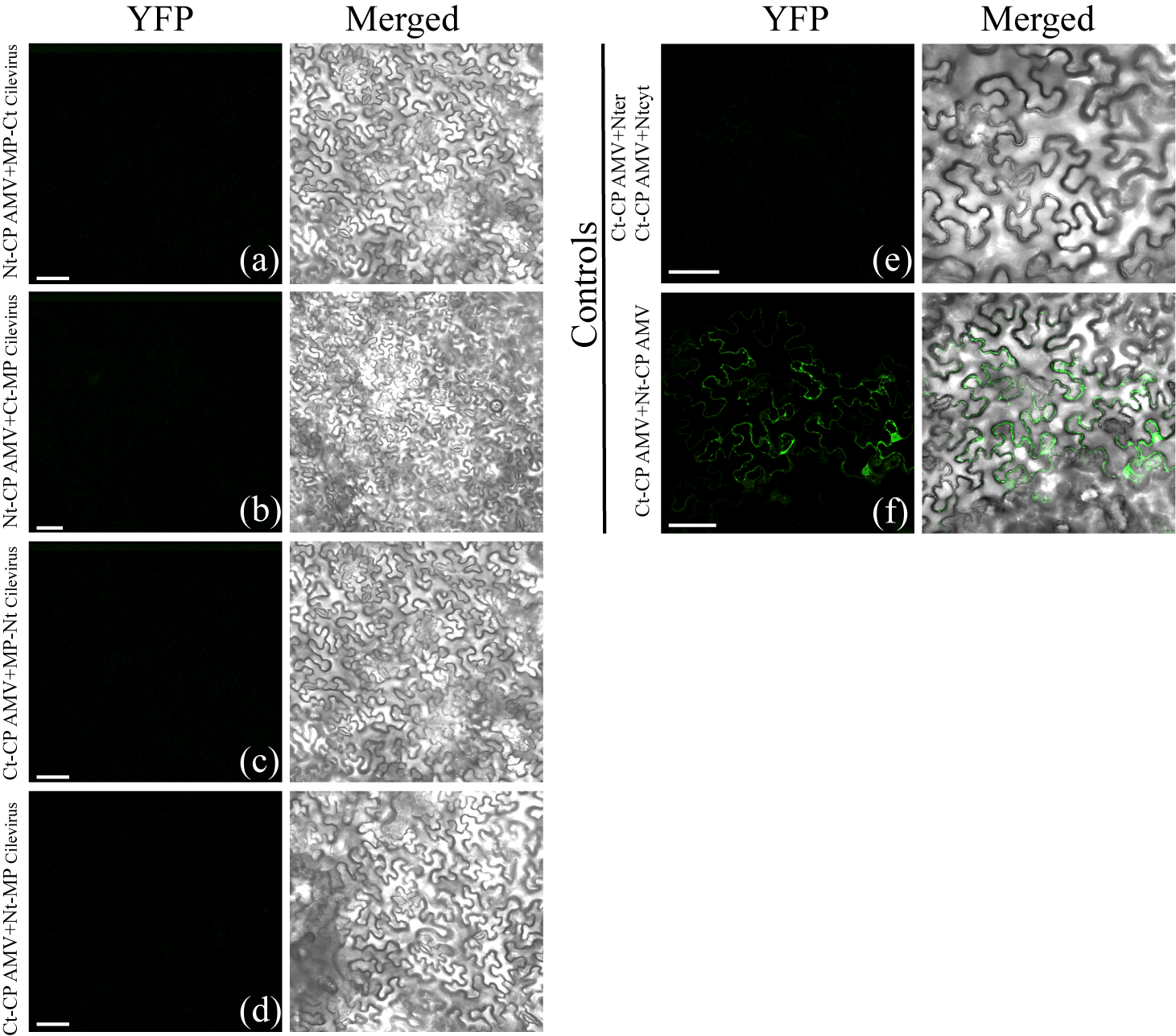


**Fig S1. BiFC assay indicate that the cileviruses MPs do not associate *in vivo* with the AMV coat protein.** BiFC analysis of heterologous interaction between AMV CP and cileviruses MPs. AMV CP carrying YFP fragments fused at their N- or C-termini (NYFP-CP and CYFP-CP) and CiLV-C MP carrying the YFP counterpart fused at their C- or N-termini were transiently co-expressed in *N. benthamiana* leaves by agroinfiltration. The protein pair combinations are shown to the left of each image. (a-d) correspond to negative interactions. Negative control corresponds to the expression of the AMV CP in combination with N-cyt or ER BiFC vector (e). BiFC positive control correspond to the dimerization of CP (NYFP-CP + CYFP-CP) (f). The positive interaction between AMV CP with its cognate MP has been previously reported ^5^. The controls of MP are shown in Fig 7. Bars correspond to 50 μm.


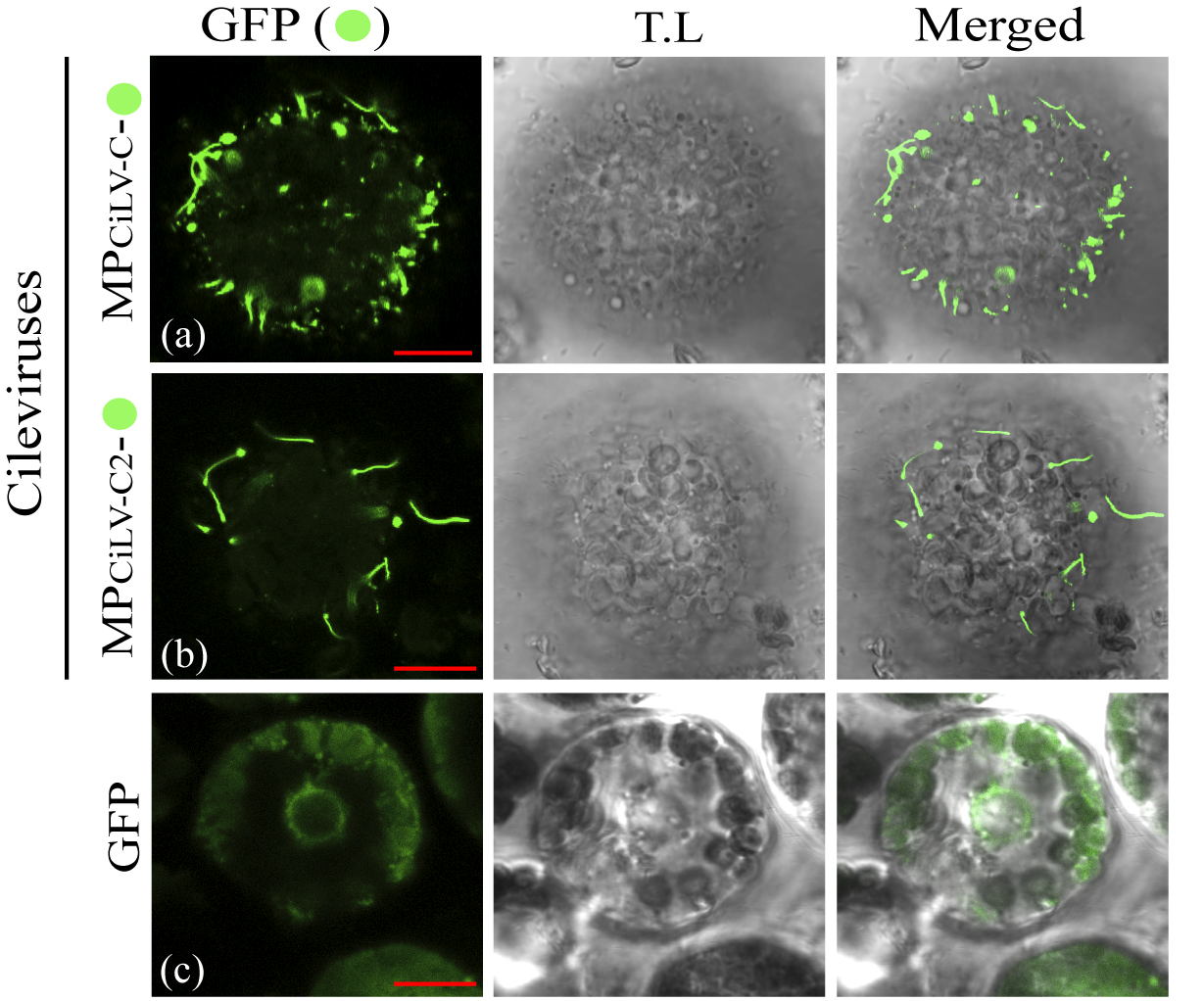


**Fig S2. The MP of cileviruses induce tubular structures on protoplast.** Analysis of tubule formation of *N. benthamiana* protoplasts transiently expressing the MP genes of CiLV-C and CiLV-C2 fused at their C-terminal with the eGFP (●). Three infiltrated leaves per construct were used for protoplasts isolation. Protoplasts were purified after one day post-infiltration and the fluorescence GFP signal was captured 16 hours post-protoplasts purification with a Zeiss LSM 780 confocal laser-scanning microscope. The green (GFP), transmitted light (TL) channels and merged images are shown. Each image-frame expressing GFP represents the visualization of several protoplasts (about 15 to 20) per assay for each MP protein analyzed. Tubule formation is observed from expression of the CiLV-C (a) and CiLV-C2 MPs (b). Diffuse GFP signal on surface of protoplast is visualized from sole GFP expression (c). Red bars correspond to 10 μm.

**
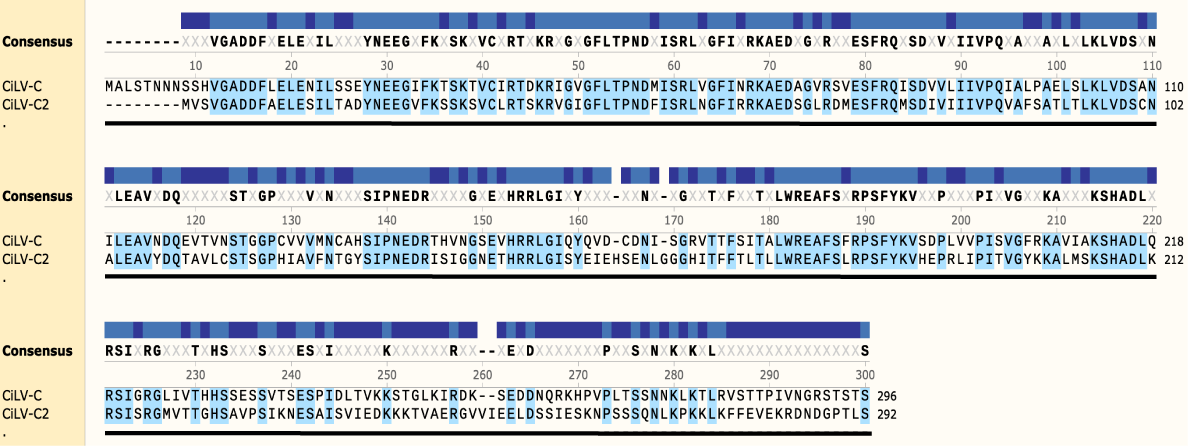
**

**Fig S3. The MP amino acids sequence alignment of CiLV-C and CiLV-C2.** Residues common at the two sequences are shown in blue (amino acids letters) and light blue (bar), whereas dissimilar residues are shown in white amino acids letters or dark blue (bar). The numbers on the right indicate the total size of the MP proteins. Virus acronyms are shown to the left of the image. The amino acids consensus is shown. The alignment was performed using the software SnapGene version 4.3.10.


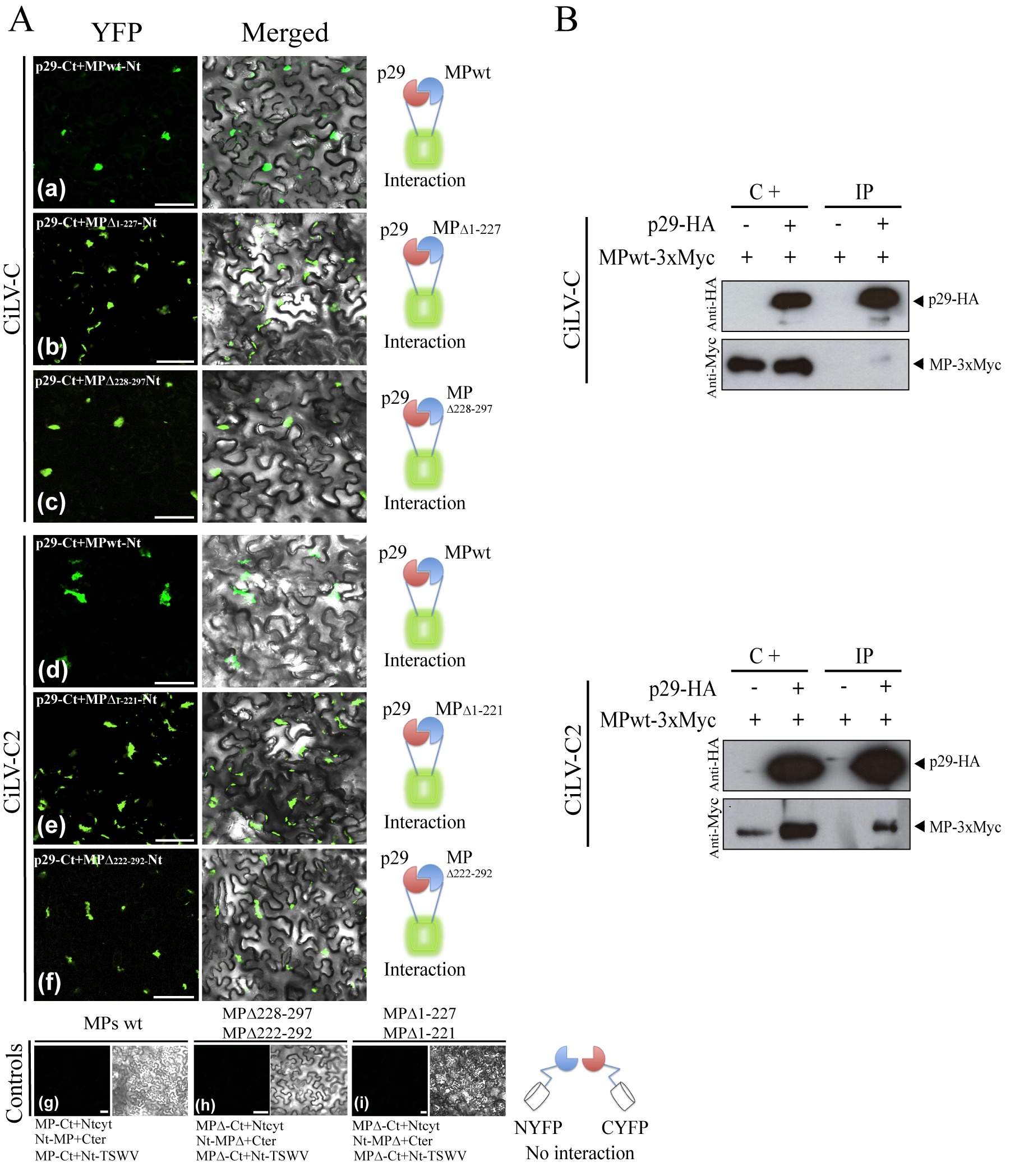


**Fig S4. Analysis of the interaction between cileviruses MPs with the cognate coat proteins (p29).** **(A)** BiFC analysis of wt and truncated MPs with the cognate coat protein (p29) of CiLV-C-and CiLV-C2. The wild-type (CiLV-C MP wt and CiLV-C2 MP wt) and the mutated MPs lacking the C-terminus (CiLV-C MPΔ_228-297_ and CiLV-C2 MPΔ_222-292_) and the remaining N-terminus (CiLV-C MPΔ_1-227_ and CiLV-C2 MPΔ_1-221_) were fused at their C-termini with the NYFP, and the correspondent cognates p29 proteins (CiLV-C p29 and CiLV-C2 p29) were also fused at their C-termini with the counterpart CYFP. This fusion protein pair combination assayed represent the combination that best expresses YFP fluorescence from all combinations previously tested by ^8^. Agrobacterium cultures transformed with the BiFC constructs were transiently co-expressed in *N. benthamiana* leaves and fluorescence visualized at four days post-infiltration. All images contain two pictures corresponding to the YFP signal or merged with transmitted light. YFP fluorescence return was detected in all situations (a-f). Negative controls correspond to the expression of the CiLV-C proteins in combination with Cyt and ER BiFC vectors or in combination with the nucleocapsid (N) protein of the tomato spotted wilt virus (TSWV) (g-i). The images displayed are representative of at least three independent experiments. The right side of each image has a representative scheme of the positive or negative interaction corresponding to all proteins assayed. Bars correspond to 50 μm. **(B)** Co-immunoprecipitation of CiLV-C and CiLV-C2 MPs with the cognate p29. Agrobacteria cultures carrying the expression cassettes for the p29-HA and MPwt-3xMyc proteins, were co-infiltrated into *N. benthamiana* leaves. At 3 days post infiltration, the immunoprecipitation of the p29-HA-tagged proteins was addressed by Pierce magnetic HA-tag kit. HA and Myc antibodies were used in the western blot analysis. The + and – signs indicate the presence or absence of the corresponding proteins in the leaf extracts. C+ and IP are referred to the positive control of expressed proteins or the immunoprecipitated proteins, respectively.

**SUPPLEMENTARY INFORMATION FIGURES 3 D, 7 C and S4 B.**


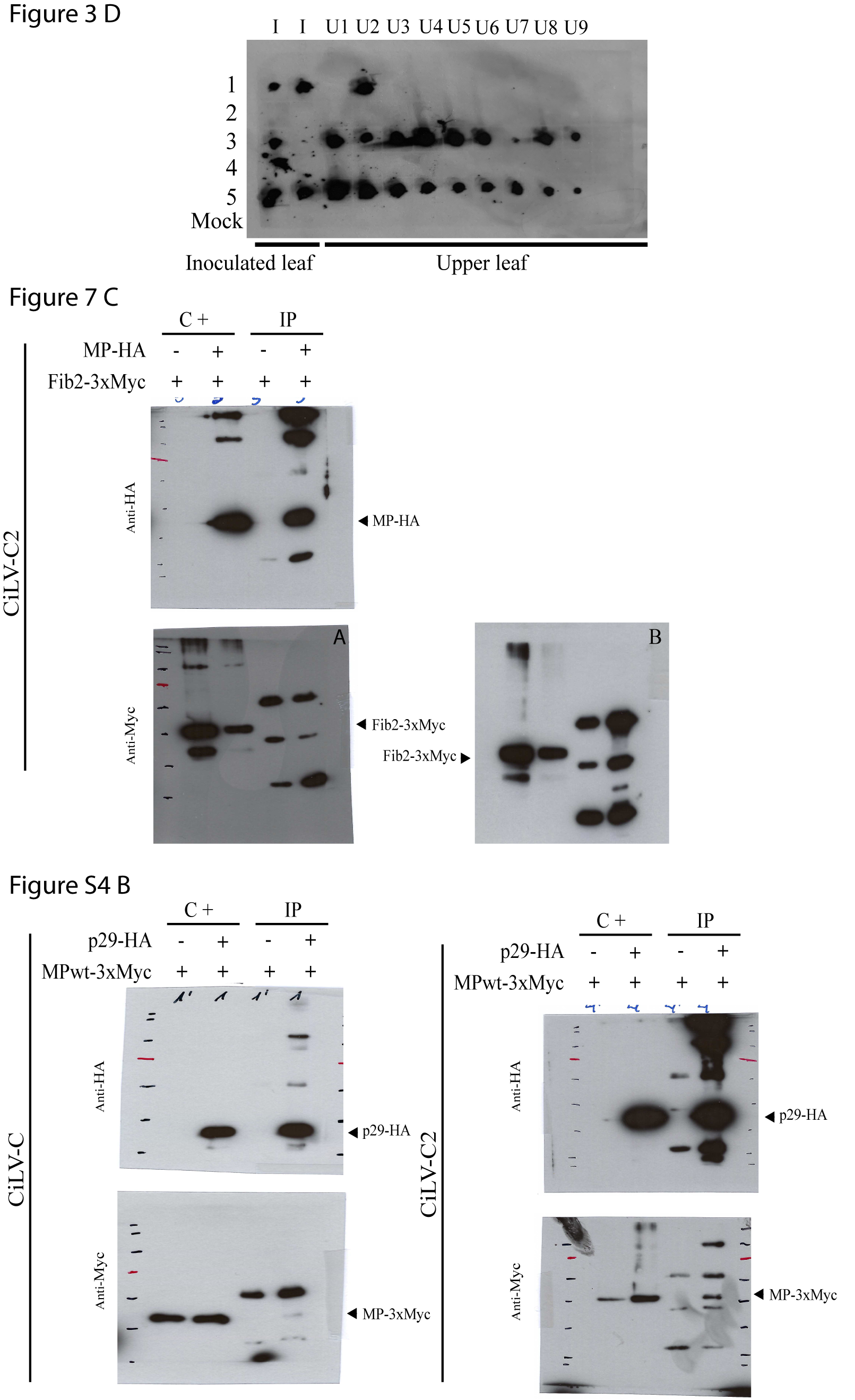


**Figure 3 D.** Tissue-printing analysis of P12 plants inoculated with a variant of AMV RNA 3 expressing the CiLV-C wt (297)(1), CiLV-C Δ_228-297_ (V)(2), CiLV-C2 wt (292)(3), CiLV-C2 Δ_222-292_ (T)(4) and AMV MP (AMV)(5). Plants were analyzed at 14 dpi by printing the transversal section of the corresponding petiole from inoculated (I) and upper (U) leaves. **Figure 7 C.** Co-immunoprecipitation of CiLV-C2 MP with Fib2. Agrobacteria cultures containing MP-HA and Fib2-3xMyc plasmids, were co-infiltrated into *N. benthamiana* leaves and extracts were analyzed at 3 days post infiltration. HA and Myc antibodies were used in the western blots. A, leaf extract treated with a non-denaturing buffer; B, leaf extract treated with RIPA buffer; C+, positive controls (samples non-immunoprecipitated) and IP, immunoprecipitated samples. The + and – signs indicate the presence or absence of the corresponding proteins in the leaf extracts. **Figure S4 B.** Co-immunoprecipitation of CiLV-C and CiLV-C2 MPs with the cognate p29. Agrobacteria cultures carrying the expression cassettes for the p29-HA and MPwt-3xMyc proteins, were co-infiltrated into *N. benthamiana* leaves. At 3 days post infiltration, the immunoprecipitation of the p29-HA-tagged proteins was addressed by Pierce magnetic HA-tag kit. HA and Myc antibodies were used in the western blot analysis. The + and – signs indicate the presence or absence of the corresponding proteins in the leaf extracts. C+ and IP are referred to the positive control of expressed proteins or the immunoprecipitated proteins, respectively.

**REFERENCES**

1 Sanchez-Navarro, J., Miglino, R., Ragozzino, A. & Bol, J. F. Engineering of alfalfa mosaic virus RNA 3 into an expression vector. *Arch Virol* **146**, 923-939 (2001).

2 Aparicio, F., Pallas, V. & Sanchez-Navarro, J. Implication of the C terminus of the Prunus necrotic ringspot virus movement protein in cell-to-cell transport and in its interaction with the coat protein. *J Gen Virol* **91**, 1865-1870, doi:10.1099/vir.0.019950-0 (2010).

3 van der Vossen, E. A., Neeleman, L. & Bol, J. F. Role of the 5' leader sequence of alfalfa mosaic virus RNA 3 in replication and translation of the viral RNA. *Nucleic acids research* **21**, 1361-1367 (1993).

4 Tenllado, F. & Bol, J. F. Genetic dissection of the multiple functions of alfalfa mosaic virus coat protein in viral RNA replication, encapsidation, and movement. *Virology* **268**, 29-40 (2000).

5 Sanchez-Navarro, J. A., Carmen Herranz, M. & Pallas, V. Cell-to-cell movement of Alfalfa mosaic virus can be mediated by the movement proteins of Ilar-, bromo-, cucumo-, tobamo- and comoviruses and does not require virion formation. *Virology* **346**, 66-73, doi:10.1016/j.virol.2005.10.024 (2006).

6 Herranz, M. C., Sanchez-Navarro, J. A., Aparicio, F. & Pallas, V. Simultaneous detection of six stone fruit viruses by non-isotopic molecular hybridization using a unique riboprobe or 'polyprobe'. *Journal of virological methods* **124**, 49-55, doi:10.1016/j.jviromet.2004.11.003 (2005).

7 Leastro, M. O., Pallas, V., Resende, R. O. & Sanchez-Navarro, J. A. The movement proteins (NSm) of distinct tospoviruses peripherally associate with cellular membranes and interact with homologous and heterologous NSm and nucleocapsid proteins. *Virology* **478**, 39-49, doi:10.1016/j.virol.2015.01.031 (2015).

8 Leastro, M. O., Kitajima, E. W., Silva, M. S., Resende, R. O. & Freitas-Astua, J. Dissecting the Subcellular Localization, Intracellular Trafficking, Interactions, Membrane Association, and Topology of Citrus Leprosis Virus C Proteins. *Frontiers in plant science* **9**, 1299, doi:10.3389/fpls.2018.01299 (2018).

9 Zamyatnin, A. A. *et al.* Assessment of the integral membrane protein topology in living cells. *Plant J* **46**, 145-154, doi:10.1111/j.1365-313X.2006.02674.x (2006).

10 Aparicio, F., Sanchez-Navarro, J. A. & Pallas, V. In vitro and in vivo mapping of the Prunus necrotic ringspot virus coat protein C-terminal dimerization domain by bimolecular fluorescence complementation. *J Gen Virol* **87**, 1745-1750, doi:10.1099/vir.0.81696-0 (2006).

11 Gomez, G. & Pallas, V. A peptide derived from a single-modified viroid-RNA can be used as an "in vivo" nucleolar marker. *Journal of virological methods* **144**, 169-171, doi:10.1016/j.jviromet.2007.04.009 (2007).

12 Genovés, A., Pallás, V. & Navarro, J. A. Contribution of topology determinants of a viral movement protein to its membrane association, intracellular traffic, and viral cell-to-cell movement. *Journal of virology* **85**, 7797-7809, doi:10.1128/JVI.02465-10 (2011).

13 Loesch-Fries, L. S., Halk, E. L., Nelson, S. E. & Krahn, K. J. Human leukocyte interferon does not inhibit alfalfa mosaic virus in protoplasts or tobacco tissue. *Virology* **143**, 626-629 (1985).

14 van Dun, C. M., van Vloten-Doting, L. & Bol, J. F. Expression of alfalfa mosaic virus cDNA1 and 2 in transgenic tobacco plants. *Virology* **163**, 572-578 (1988).

15 Taschner, P. E., Van der Kuyl, A. C., Neeleman, L. & Bol, J. F. Replication of an incomplete alfalfa mosaic virus genome in plants transformed with viral replicase genes. *Virology* **181**, 445-450 (1991).

16 Dunn, K. W., Kamocka, M. M. & McDonald, J. H. A practical guide to evaluating colocalization in biological microscopy. *American journal of physiology. Cell physiology* **300**, C723-742, doi:10.1152/ajpcell.00462.2010 (2011).
